# Genome-Wide Exploration of Thiamin Pyrophosphate Riboswitches in Medically Relevant Fungi Reveals Diverse Distribution and Implications for Antimicrobial Drug Targeting

**DOI:** 10.1021/acsomega.4c00158

**Published:** 2024-12-10

**Authors:** Valdemir Vargas-Junior, Ana Carolina Ramos Guimarães, Ernesto Raul Caffarena, Deborah Antunes

**Affiliations:** 1 Laboratory for Applied Genomics and Bioinnovations, Oswaldo Cruz Institute (IOC - FIOCRUZ), Rio de Janeiro 21040-900, Brazil; 2 Computational Biophysics and Molecular Modeling Group, Scientific Computing Program (PROCC - FIOCRUZ), Rio de Janeiro 21040-360, Brazil

## Abstract

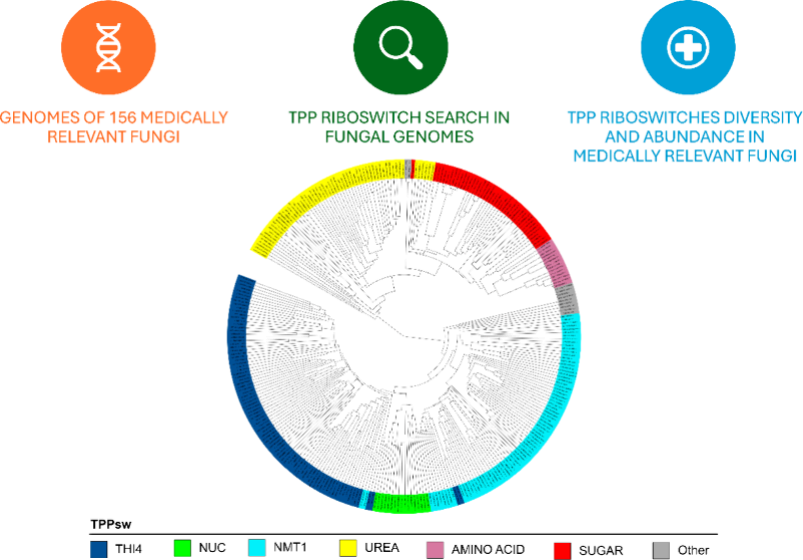

The rising incidence of fungal infections coupled with limited treatment options underscores the urgent need for novel antifungal therapies. Riboswitches, particularly thiamin pyrophosphate (TPP) class, have emerged as promising antimicrobial targets. This study presents a comprehensive genome-wide analysis of TPP riboswitches in 156 medically relevant fungi utilizing advanced covariance models (CMs) tailored for fungal sequences. Our investigation identified 378 conserved TPP riboswitch sequences distributed across 140 distinct species, revealing a broader prevalence than that previously recognized. Notably, we provide evidence for a novel putative group of TPP riboswitches, designated TPPsw^SUGAR^, associated with sugar transporters in Mucoromycota and Basidiomycota. This group exhibits distinctive structural features while maintaining key TPP-binding motifs, potentially expanding our understanding of the riboswitch diversity in fungi. Our analysis highlights the impact of P3 stem variability on riboswitch detection and characterization, demonstrating the superiority of fungal-specific CMs over generic models. We observed multiple TPP riboswitches in over 50% of the examined species, including clinically significant pathogens involved in aspergillosis and mucormycosis. Remarkably, *Aspergillus latus*, a species associated with COVID-19 coinfections, harbors six distinct TPP riboswitch sequences, whereas the extremophilic black fungus *Hortaea werneckii* possesses nine. These findings not only elucidate the diverse distribution of TPP riboswitches in pathogenic fungi but also emphasize their potential as multifaceted targets for antifungal drug development. By addressing key limitations of previous detection methods and providing insights into riboswitch structural diversity, this study lays a foundation for future investigations into riboswitch-mediated regulation in fungi and the development of novel antifungal strategies.

## Introduction

The incidence of fungal infections in humans has grown alarmingly in recent years.^1^ These infections affect over 300 million individuals globally, with an estimated annual mortality of 1.6 million.^2,3^

Notably, certain fungal infections have garnered significant international attention during the Covid-19 pandemic, as they have been identified as coinfections in patients with this viral disease.^4−18^ The list of fungal coinfections related to Covid-19 encompasses aspergillosis, candidiasis, cryptococcosis, mucormycosis, pneumocystosis, and several endemic mycoses.^4−7,10,13−19^ Among these, aspergillosis and mucormycosis have emerged as substantial diseases with alarming mortality rates.^4,14,16,20^ Even before the pandemic, chronic pulmonary aspergillosis affected more than 3 million people each year.^2^ This infection is caused by various species within the *genus Aspergillus*, with *Aspergillus fumigatus* being the most frequently associated pathogen.^8,19,21−27^ Unfortunately, this challenging scenario is compounded by limited availability of effective antifungal therapies and the emergence of drug-resistant fungal strains.^25,28,29^ As aggravating factors, most currently available antifungal drugs rely on only a few primary mechanisms. This means that when a fungus develops resistance to one of them, it significantly reduces the treatment options.^30−38^ In fact, some species, such as *Lomentospora prolificans*, have already exhibited resistance to most currently available antifungal drugs.^38−42^ Additionally, current antifungal therapies often lead to significant side effects due to the similarity between human and fungal cells. Therefore, the need of developing novel antifungal therapies becomes evident.^30,31,43−49^

To address the global burden of fungal infections, the World Health Organization (WHO) released a priority list of fungal pathogens, highlighting the urgent need for new therapies.^50^ This initiative echoes a comparable undertaking in 2017 when the WHO released a priority list for bacterial pathogens.^51^

Riboswitches are molecular structures present in the introns and untranslated regions (UTRs) of the mRNA (mRNA) of several species of bacteria, archaea, fungi, and plants.^52,53^ These structures play a crucial role in the recognition and post-transcriptional regulation of specific metabolites including vitamins, ions, and nitrogenous bases.^54,55^ The riboswitch consists of an evolutionarily conserved aptamer domain associated with the expression platform.^56−58^ The fundamental principle of the riboswitch function relies on two structurally stable conformations that can interchange depending on the presence or absence of their respective ligands in their binding site.^54,59,60^ Among the over 40 classes of known riboswitches, the thiamin pyrophosphate (TPP) riboswitch appears to be the only class of riboswitches found in fungi.^57,61−65^

The TPP riboswitch, a type of riboswitch that recognizes and binds to TPP, is the most prevalent riboswitch class found in three domains of life: Archaea, Bacteria, and Eukarya.^56,66,67^ TPP is an essential cofactor for several organisms, participating in central metabolic pathways, such as the Krebs cycle and pentose-phosphate pathway.^68−71^ Consequently, TPP plays a crucial role in the growth and survival of many organisms,^71−73^ making the TPP riboswitch an intriguing target for the development of novel antimicrobial drugs. Nonetheless, the antimicrobial potential of TPP analogs in bacteria and fungi has only been demonstrated in a limited number of studies,^66,74,75^ whereas others have provided confirmatory evidence.^63^

Thus, far, five groups of TPP riboswitches have been reported in fungi.^61,62,65^ In the first two, the TPP riboswitch regulates thi4 (TPPsw^THI4^) and nmt1 (TPPsw^NMT1^) genes associated with the biosynthesis of the two moieties constituting TPP. In the three others, the TPP riboswitch is believed to control the gene expression of distinct families of transporter proteins: urea (TPPsw^UREA^), nucleoside (TPPsw^NUC^), and amino acid (TPPsw^AA^) transporters. Previous studies also found other target genes associated with riboswitch sequences, suggesting the possibility of additional groups of TPP riboswitches in fungi that could not be classified at that time.^61,65^

Certain fungal species have multiple TPP riboswitches within their genomes.^61,65^ Moreover, the TPP riboswitch exhibits remarkable conservation across species, particularly regarding its secondary and tertiary structures. In our recent study, which focused on three distinct TPP riboswitches discovered in *Aspergillus oryzae*, we revealed similarities in TPP dynamics and binding free energy.^63^ These findings highlight the importance of gaining comprehensive understanding of their functional characteristics.

Given the potential use of TPP riboswitches as drug targets and their involvement in critical post-transcriptional processes, it is imperative to explore their distribution and characteristics in medically relevant fungal species. In this study, we conducted a comprehensive search for TPP riboswitches in the genomes of 156 fungal pathogens, encompassing all fungal groups in the WHO’s priority list, as well as other fungal species associated with relevant infections in humans.^2,4−6,18,28,29,50,76−84^ In addition, we conducted a comparative analysis of riboswitches from these species using multiple covariance models (CMs) to augment the precision of TPP riboswitch detection. By elucidating the presence and diversity of TPP riboswitches in medically important fungi, we aim to contribute to the development of novel antifungal therapies with improved efficacy against these infections.

## Results

### TPP Riboswitch Distribution in Fungal Pathogens

Our comprehensive analysis of 156 medically relevant fungal species revealed a widespread distribution of TPP riboswitches, with 140 species (89%) harboring at least one regulatory element. A total of 378 TPP riboswitch sequences were identified, of which 322 were assigned to previously known riboswitch groups. Notably, 116 species (74%) possessed two or more TPP riboswitches, highlighting the prevalence of multiple regulatory elements within the individual genomes.

The distribution of TPP riboswitches across different groups was as follows: 107 sequences of TPPsw^THI4^, 91 sequences of TPPsw^NMT1^, 81 sequences of TPPsw^UREA^, 26 sequences of TPPsw^NUC^, and 18 sequences of TPPsw^AA^. Additionally, we identified 43 sequences associated with sugar transporters, providing evidence for a potentially new group of fungal TPP riboswitches, which we designated as TPPsw^SUGAR^. The remaining 12 identified sequences were associated with other target genes that could not be included in any of the established TPP riboswitch groups.

**Figure 1:**
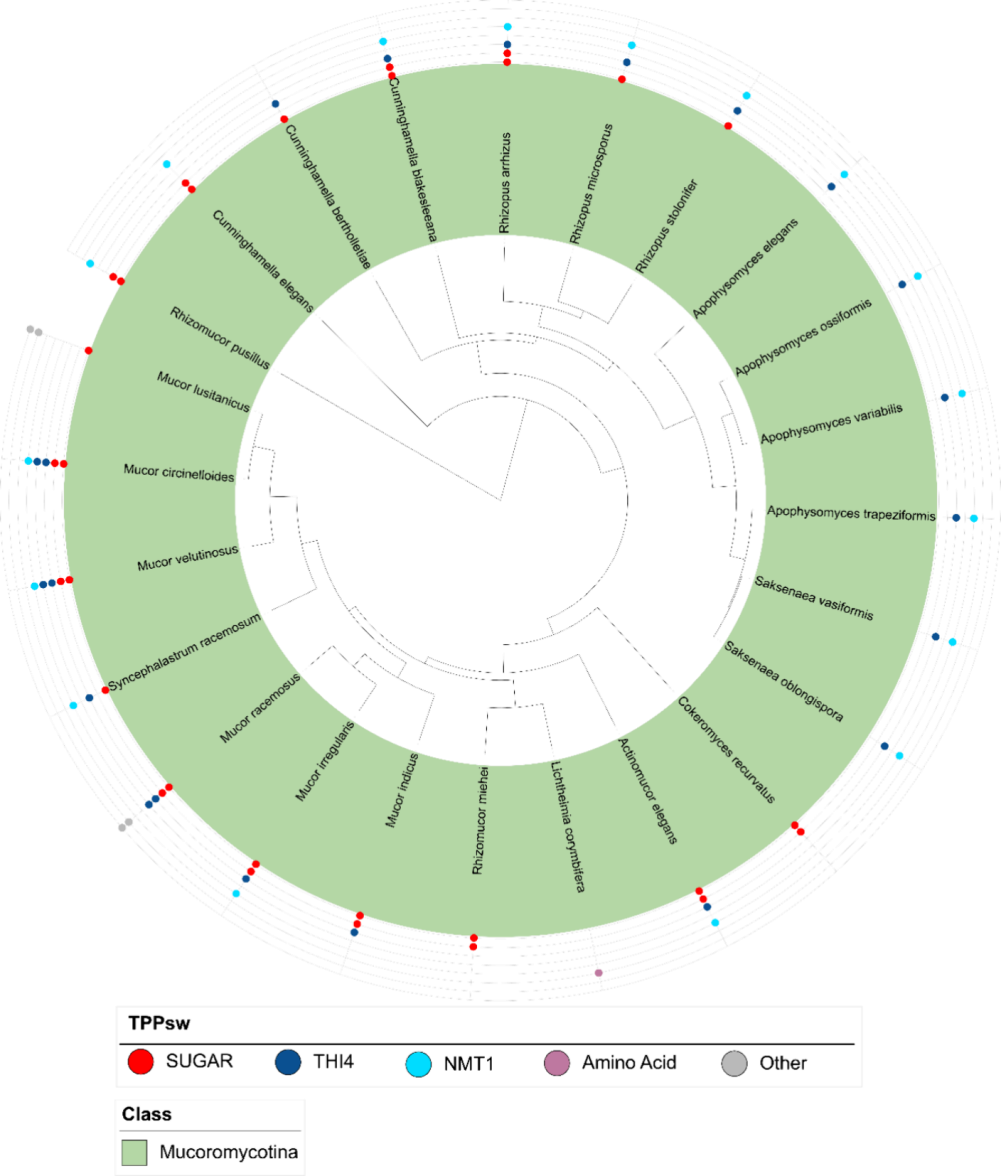
Distribution and diversity of TPP riboswitches across Mucoromycota species. The phylogenetic tree shows the relationships among the Mucoromycota species, with Mucoromycotina highlighted in green. Colored circles represent different TPP riboswitch groups identified in each species as defined in the legend. Circle quantity indicates the number of detected riboswitches. This visualization revealed the prevalence of TPPsw^SUGAR^ (red), TPPsw^THI4^ (blue), and TPPsw^NMT1^ (cyan) in Mucoromycota, and demonstrated the diversity of TPP riboswitch types across the phylum.

Interestingly, the TPPsw^SUGAR^ group exhibited a remarkable prevalence in Mucoromycota species, emerging as the most abundant group within this taxon (Figure 1). This distribution pattern suggests a specialized role for the TPP-based regulation of sugar transport in these fungi. Notably, other transporter families were largely absent in most Mucoromycotina species, except for the TPPsw^AA^ group found in *Lichtheimia corymbifera*.

Our analysis also revealed the co-occurrence of two TPP riboswitches that regulate TPP biosynthesis in multiple Mucormycota species, indicating a potentially more complex regulatory network in these fungi. In *Mucor irregularis* and *Mucor lusitanicus*, we identified proteins annotated as ATP-binding domains related to ABC transporters. However, further examination using deep TMHMM revealed the absence of transmembrane domains in these proteins, suggesting that they are unlikely to be involved in membrane transport processes.

This diverse distribution of TPP riboswitches across different functional groups underscores their importance in regulating various aspects of fungal metabolism, from thiamine biosynthesis to transport of essential molecules. The identification of the putative TPPsw^SUGAR^ group, particularly prevalent in Mucoromycota species, suggests a previously unrecognized role of TPP-based regulation in fungal sugar transport.

### Distribution of TPP Riboswitches in Basidiomycota and Ascomycota

**Figure 2:**
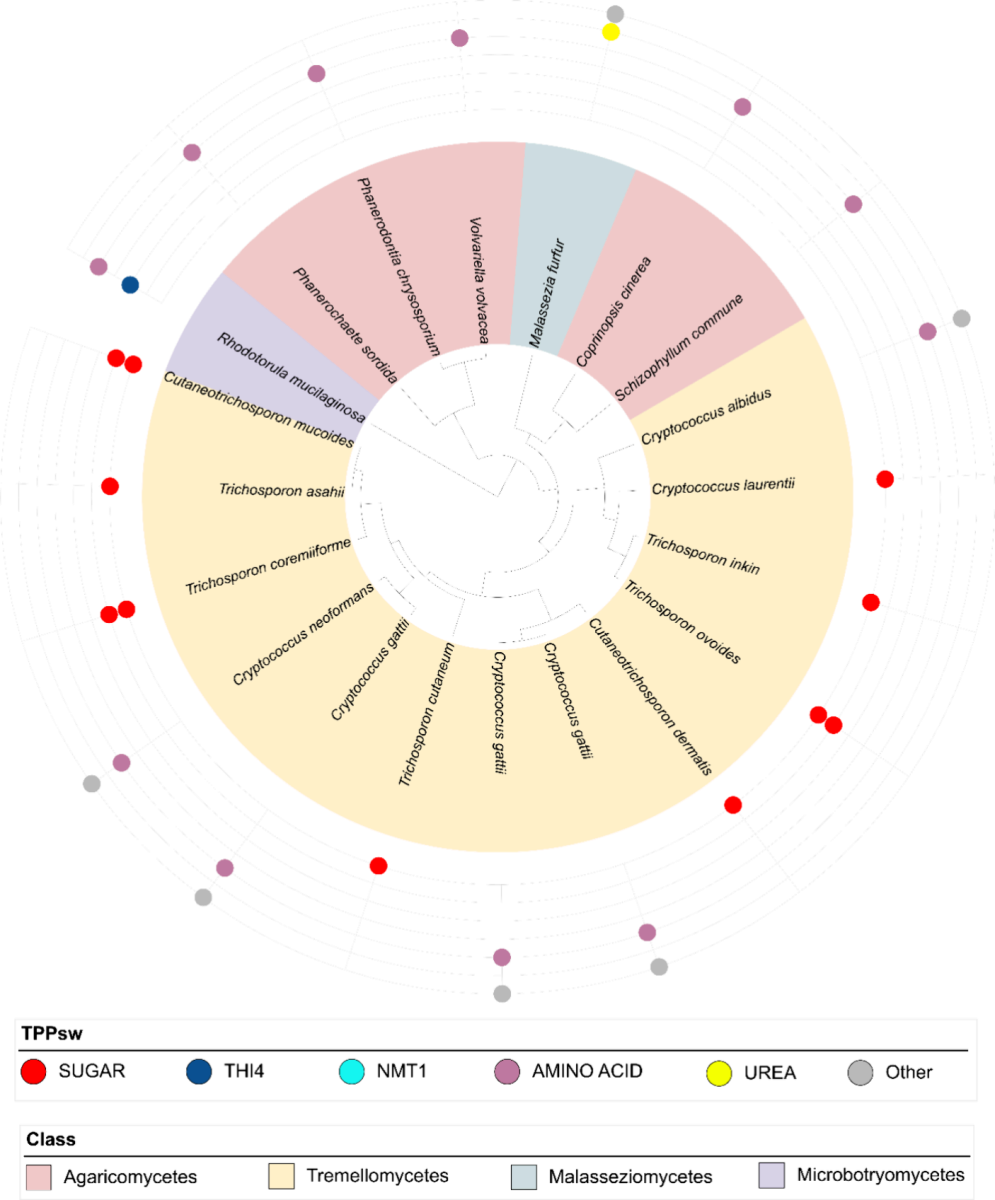
TPP riboswitch distribution in Basidiomycota. The tree depicts the phylogenetic relationships among Basidiomycota species, with classes indicated by colors in the legend. Colored circles represent the different TPP riboswitch groups found in each species, as defined in the legend. The quantity of circles corresponds to the number of detected riboswitches. This figure highlights the predominance of TPPsw^AA^ (pink) and TPPsw^SUGAR^ (red) in Basidiomycota, and illustrates the varying distribution of TPP riboswitch types across the phylum.

According to our analysis, the most prevalent TPP riboswitch group In Basidiomycota, was TPPsw^AA^ (Figure 2), which regulates amino acid transporters.

These transporters are typically responsible for the uptake of various amino acids, including branched-chain and aromatic amino acids. Additionally, we identified a TPP riboswitch associated with a protein annotated as an amino butyric acid transporter in a few species. TPPsw^UREA^ was exclusively found in *Malassezia furfur*. Notably, TPPsw^NUC^ and TPPsw^NMT1^ were absent in Basidiomycota, except for the presence of TPPsw^THI4^ in *Rhodotorula mucilaginosa*. The prevalence of TPPsw^THI4^ and TPPsw^UREA^ was similar across the examined species. These findings suggest that TPP riboswitches have limited regulatory roles in governing TPP biosynthesis within Basidiomycota, with greater emphasis on regulating amino acid transport.

**Figure 3:**
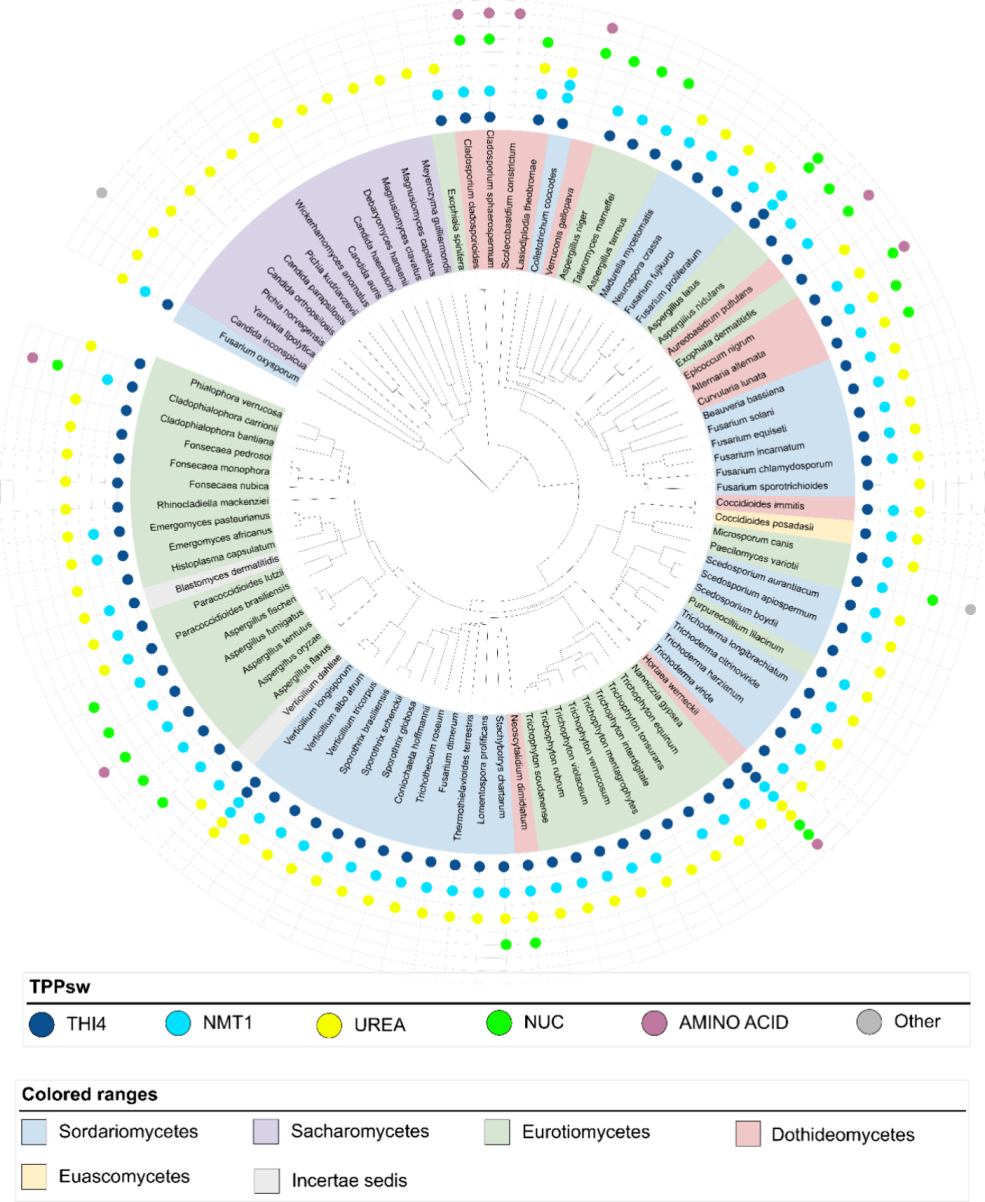
Prevalence and diversity of TPP riboswitches in Ascomycota. The phylogenetic tree shows relationships among Ascomycota species, with classes indicated by colors defined in the legend. Colored circles represent the different TPP riboswitch groups identified for each species. Circle quantity indicates the number of detected riboswitches. This visualization shows the wide distribution of TPPsw^THI4^ (blue), TPPsw^NMT1^ (cyan), and TPPsw^UREA^ (yellow) across Ascomycota, while also highlighting the absence of TPPsw^SUGAR^ in this phylum.

In Ascomycota, all TPP riboswitch groups were present, except for TPPsw^SUGAR^ (Figure 3), whereas TPPsw^UREA^ was abundant across most species. However, in some *Aspergillus* and certain Dothideomycete species, TPPsw^NUC^ was observed instead of TPPsw^UREA^. Additionally, TPPsw^THI4^ and TPPsw^NMT1^ were prevalent in Ascomycota, except for their absence in *Candida* spp.

Our bioinformatic analysis identified some Ascomycota species with a notably high number of TPP riboswitches. Among these, *Hortaea werneckii* was found to have the highest number, with a total of nine TPP riboswitches. These consisted of four pairs (two each of TPPsw^THI4^, TPPsw^NMT1^, TPPsw^NUC^, and TPPsw^UREA^) and a single TPPsw^AA^. The presence of these paired riboswitches in *H. werneckii* can be directly attributed to complete genome duplication,^85−87^ which resulted in two copies of each riboswitch-regulated gene.

Among *the Aspergillus* species, we typically detected three riboswitches: TPPsw^NMT1^, TPPsw^THI4^, and TPPsw^NUC^. However, *Aspergillus latus* stood out with six TPP riboswitches, specifically, three pairs corresponding to TPPsw^NMT1^, TPPsw^THI4^, and TPPsw^NUC^. This doubling of TPP riboswitches in *A. latus* is consistent with its known allodiploid genome structure resulting from an interspecies hybridization event. As an allodiploid species, *A. latus* contains a full set of chromosomes from each parent species, effectively doubling its genomic content, including TPP riboswitch-regulated genes.

### Characteristics of TPPsw^SUGAR^

**Figure 4:**
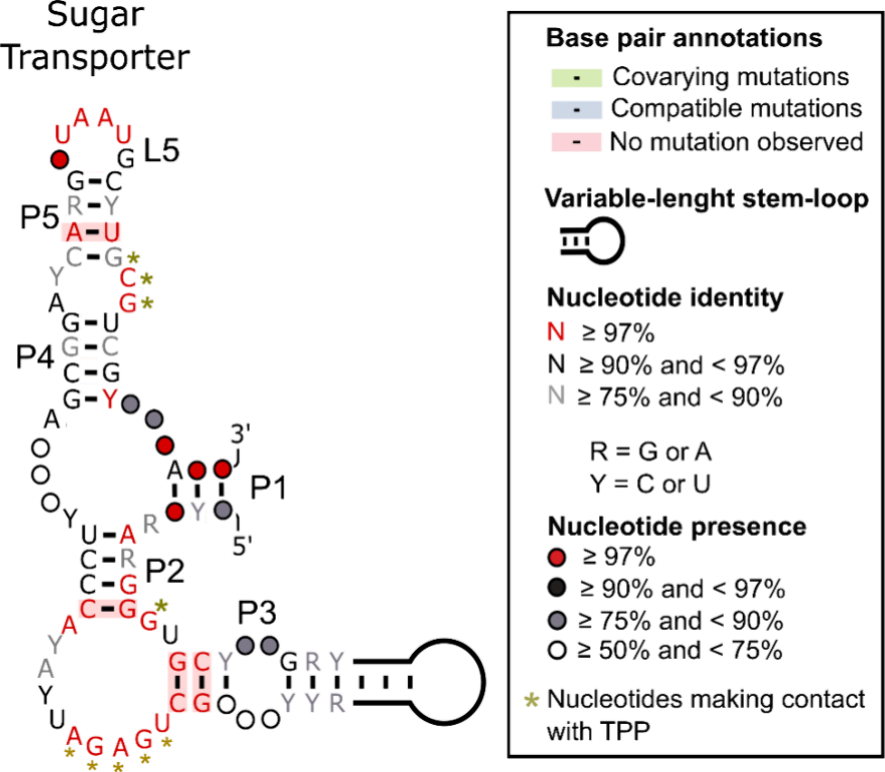
Consensus secondary structure of the putative TPPsw^SUGAR^ riboswitch. The structure is color-coded to indicate the nucleotide conservation levels, as defined in the legend. Golden asterisks mark nucleotides predicted to interact with TPP based on their structural similarity to known TPP riboswitches. The key structural elements (P1–P5, L2-L5, J2–4) are labeled. This model highlights both the conserved features shared with other TPP riboswitches and the unique structural characteristics of the TPPsw^SUGAR^ group.

Our comprehensive analysis uncovered a group of 43 sequences associated with sugar transporters that exhibit the key characteristics of TPP riboswitches while displaying distinct features. We named this group the TPPsw^SUGAR^. These sequences maintain the hallmark elements of TPP riboswitches, including highly conserved nucleotides in the J2–3 junction region, which are typically involved in binding to the pyrimidine ring of TPP (Figure 4). Furthermore, the pyrophosphate binding pocket, characterized by the conserved GCG sequence, is present and retains the residues responsible for interacting with the pyrophosphate moiety of TPP.

The overall secondary structure of the TPPsw^SUGAR^ sequences conforms to the characteristic three-way junction architecture of the TPP riboswitches. A distinguishing feature of the TPPsw^SUGAR^ group is its P3 stem, which shows notable variability compared with other fungal TPP riboswitch groups. While the average length of the P3 stem in TPPsw^SUGAR^ was shorter (115 nucleotides) than that in the other groups, it exhibited considerable sequence diversity. This variability in the P3 region, combined with differences observed in the P2 and P4 stems and the J2–4 region, contributes to the unique structural profile of the TPPsw^SUGAR^ group.

Genomic context analysis revealed that TPPsw^SUGAR^ sequences were consistently located in the 5′-UTR of genes annotated as sugar transporters across multiple fungal species. This consistent position suggests a conserved regulatory function related to sugar transport. Phylogenetically, TPPsw^SUGAR^ sequences were primarily identified in Mucoromycota, with some occurrences in Basidiomycota. This distribution pattern indicates potential lineage-specific adaptations of these regulatory elements.

Although these structural and genomic features provide evidence for a distinct group of TPP riboswitches associated with sugar transporters, it is important to note that experimental validation is crucial. Future studies, including in vitro binding assays and in vivo functional analyses, are necessary to confirm the regulatory activity of these sequences and their specificity for TPP. Such experimental work is essential to fully establish the role of TPPsw^SUGAR^ as a new functional group of TPP riboswitches in fungi.

### Structural Conservation and Impact of P3 Size on TPP Riboswitch Analysis

**Figure 5:**
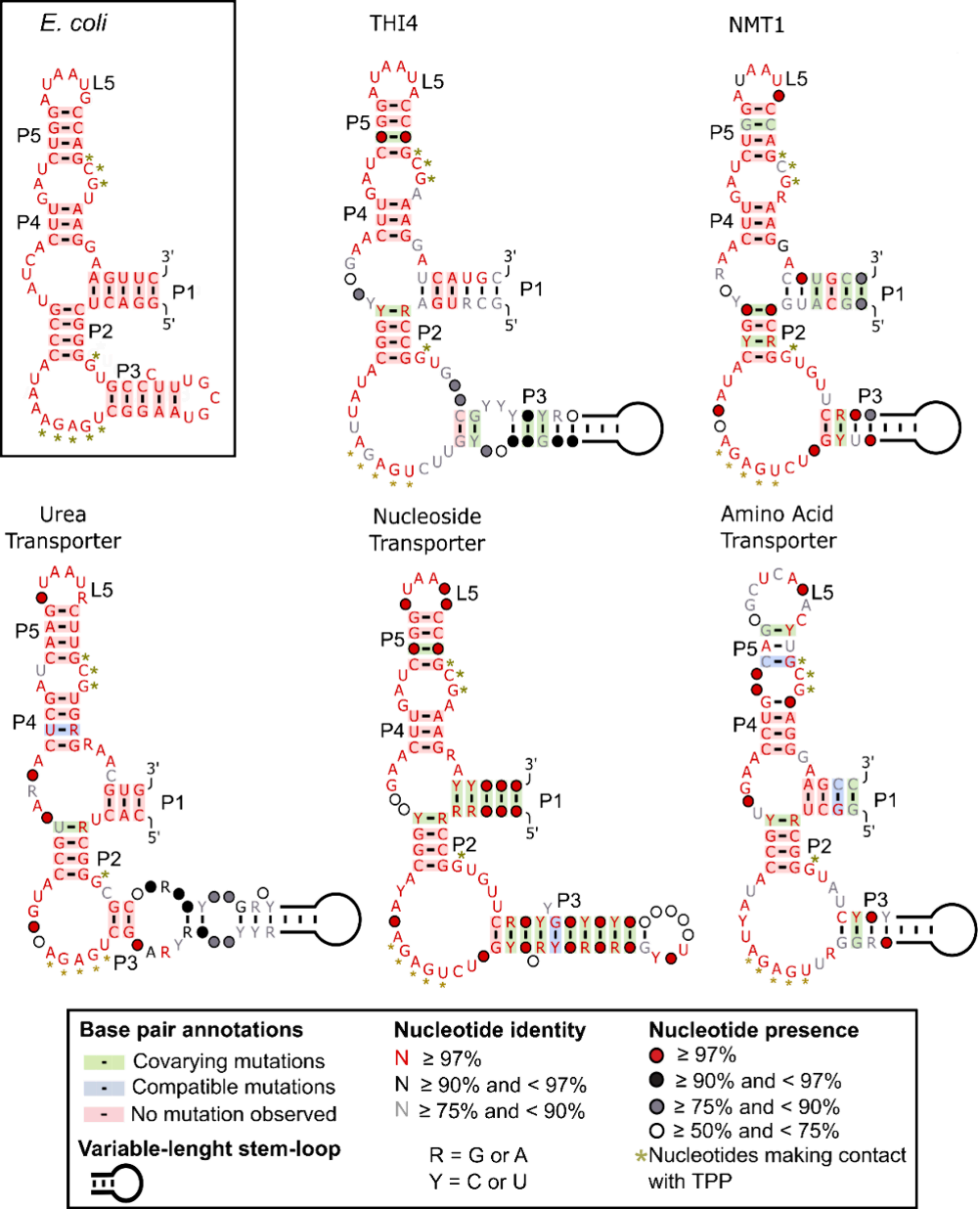
Consensus secondary structures of established fungal TPP riboswitch groups. Each structure represents a distinct TPP riboswitch group as labeled. Nucleotide conservation is indicated by color, as defined in the legend. Golden asterisks mark nucleotides predicted to interact with TPP. The experimentally validated structure of the *E. coli* ThiA riboswitch is provided as a reference. This comparison highlights both conserved elements across TPP riboswitches and group-specific structural variations.

TPP riboswitches exhibit distinct sequence and structural conservation patterns across diverse fungal species. Our analysis of the consensus secondary structures revealed a high degree of conservation in most motifs, with nucleotides crucial for TPP binding being consistently preserved (Figure 5). The P2, P4, and P5 regions demonstrated the highest conservation levels and played pivotal roles in maintaining the overall structure and function of the riboswitch.

Notably, the P3 motif exhibited significant variation in sequence identity, length, and base pairing. This variability in P3 contrasts with the more conserved nature of other structural elements. The interaction between the proximal segment of the P3 and P5 nucleotides is crucial for stabilizing the “on-state” of the TPP riboswitch, highlighting the functional importance of P3 despite its variability.

Among the established TPP riboswitch groups, TPPsw^AA^ showed lower conservation, specifically in the L5 region, and lacked the conserved sequence 5′-RUAAUR-3, ′ which is typically found in this area in other fungal TPP riboswitch groups. This distinctive feature of TPPsw^AA^ may have implications for its function and regulation, and warrants further investigation.

Our analysis revealed that the putative TPPsw^SUGAR^ group displayed the highest overall variability compared with the well-established TPP riboswitch groups (Figures 4 and 5). This group showed notable variations across multiple structural elements, particularly in the P2, P3, and P4 stems, as well as in the J2–4 region. Despite this global variability, some structural elements and nucleotides corresponding to the TPP binding sites in known riboswitches appear to be conserved. It is important to note that the apparent higher variability in the putative TPPsw^SUGAR^ group may be influenced by the relatively small sample size of the sequences currently available.

**Figure 6:**
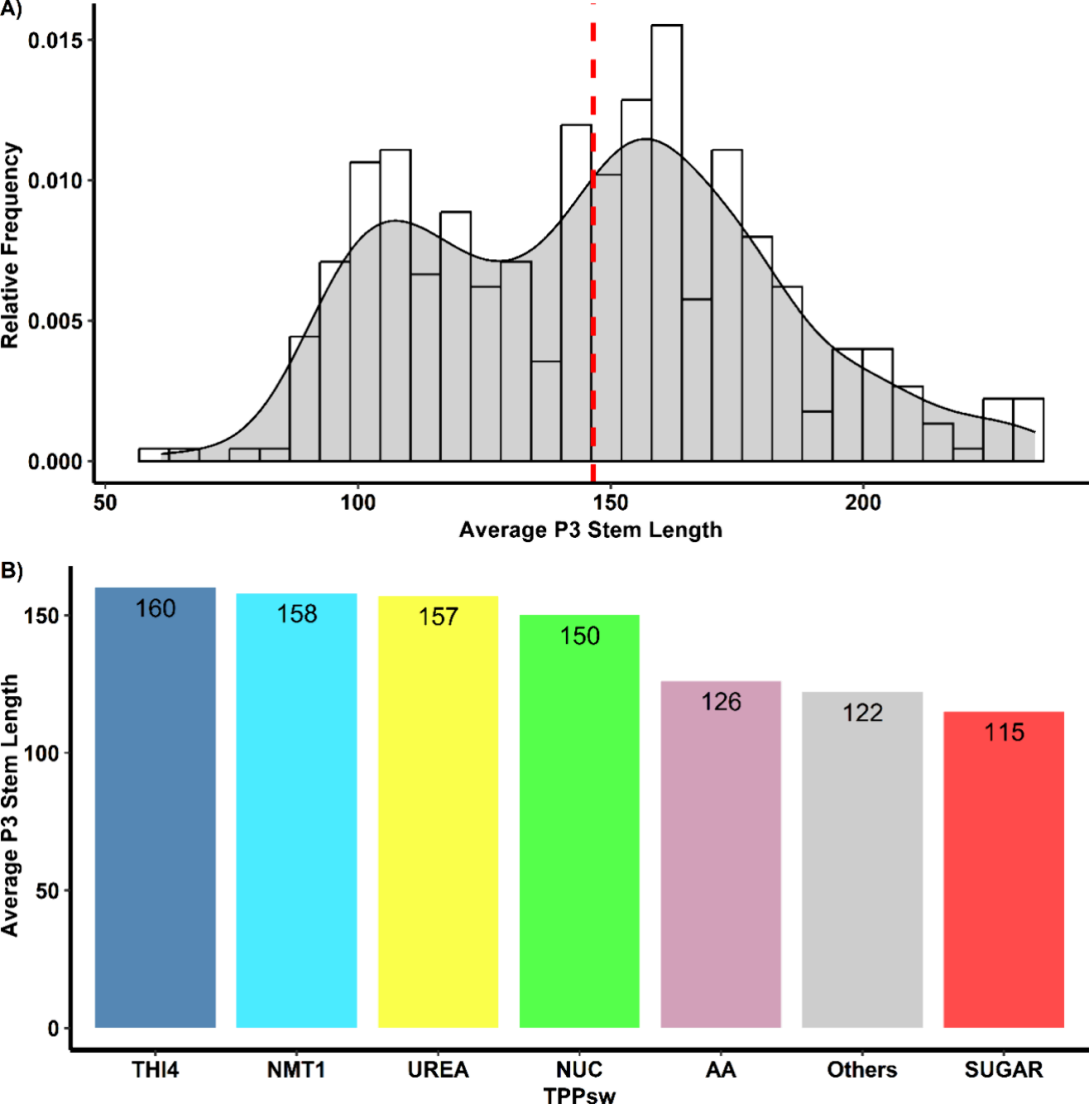
Distribution of P3 stem lengths in fungal TPP riboswitches. (A) Histogram showing the frequency distribution of P3 stem lengths across all the identified TPP riboswitches. The *y*-axis represents the relative frequency of the sequences, and the vertical red dashed line indicates the mean P3 stem length. (B) Average P3 stem length for each TPP riboswitch. Bars represent the mean P3 stem length for sequences in each group, with the exact values shown above each bar. This analysis revealed substantial variability in P3 stem length among fungal TPP riboswitches, particularly when compared with their bacterial counterparts.

The variability in P3 length significantly affects the detection and analysis of TPP riboswitches in fungi. We observed that the P3 stem in fungal TPP riboswitches exceeded the combined length of all the other aptamer regions. For instance, in *Scedosporium* species, we identified TPP riboswitches with P3 lengths reaching 172 nucleotides, whereas the total aptamer size encompassed 234 nucleotides. This is in stark contrast to bacterial TPP riboswitches, such as the *Escherichia coli* ThiA riboswitch (PDB ID: 2GDI), which has a total length of 80 nucleotides and only 18 nucleotides in the P3 region. In our data set, the average P3 length among fungal sequences was approximately 150 nucleotides (Figure 6).

This extensive variability in P3 length poses challenges for TPP riboswitch detection using standard CMs. We found that RF00059 CM from Rfam, which is built with riboswitch sequences from various domains of life, often fails to accurately identify the full sequence of fungal TPP riboswitches, particularly those with longer P3 stems. When the P3 helix exceeds a certain length threshold (typically >60 nucleotides), the output often reports an incomplete sequence, with the detected region ending at some point within the P3 stem.

To address this issue, we constructed and calibrated fungal-specific CMs using the TPPsw^NMT1^, TPPsw^THI4^, and TPPsw^UREA^ sequences. These custom CMs demonstrated improved performance in detecting TPP riboswitches in fungal genomes, including those with extended P3 regions that were missed by RF00059 CM.

**Figure 7:**
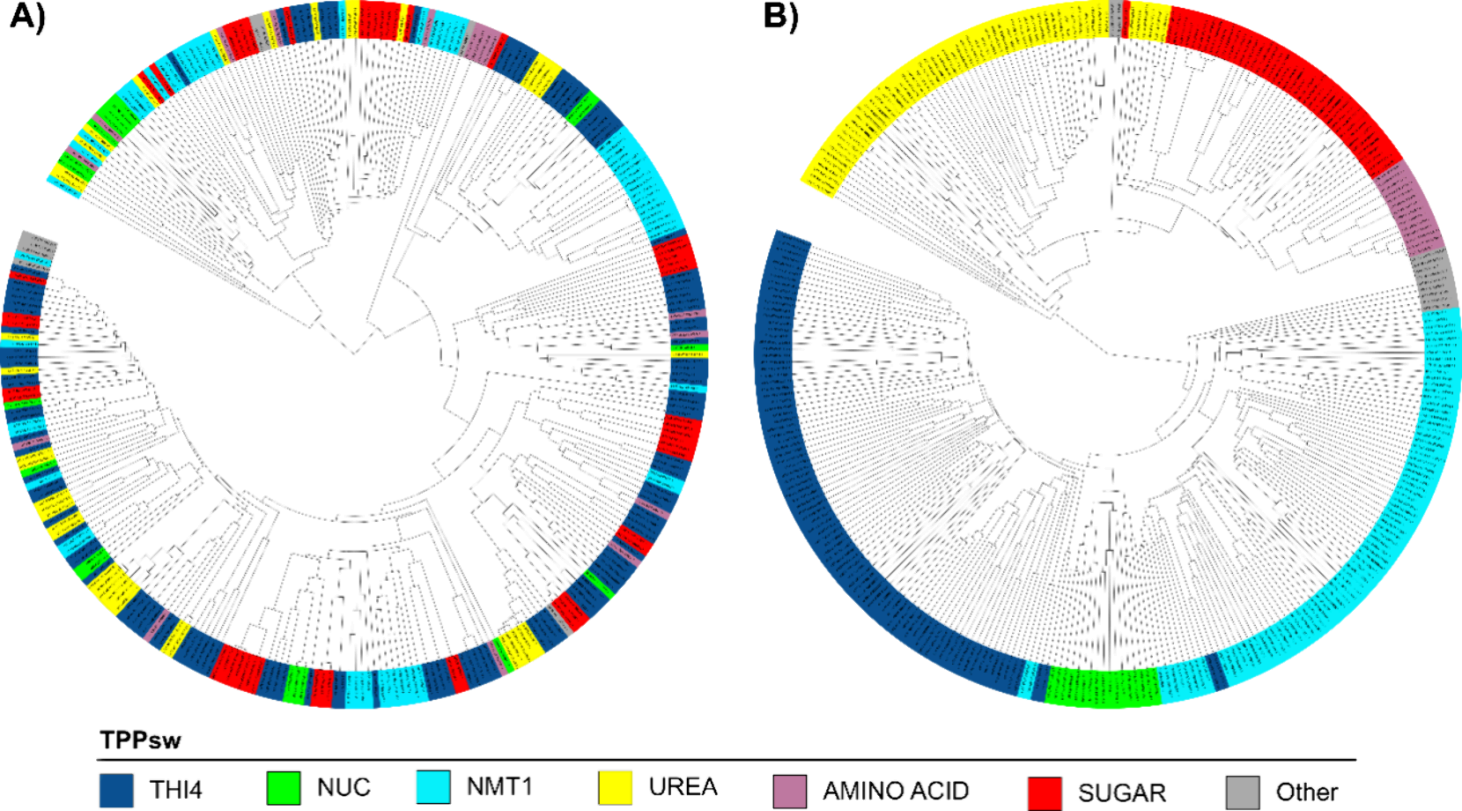
Impact of P3 stem on TPP riboswitch clustering. Trees represent hierarchical clustering of TPP riboswitch sequences based on their structural similarity. Colors indicate different TPP riboswitch groups as defined in the legend. (A) Clustering of full-length TPP riboswitch sequences. (B) Clustering of TPP riboswitch sequences with P3 stem nucleotides excluded. This comparison demonstrates that excluding highly variable P3 regions results in improved grouping of sequences according to their associated genes, highlighting the impact of P3 variability on sequence analysis and classification.

The impact of P3 variability also extends to sequence alignment and clustering. Initial clustering based on the entire riboswitch length resulted in random groupings that did not correlate with the genes regulated (Figure 7A). However, when we excluded P3 stem nucleotides from each sequence and performed reclustering, we observed distinct groupings that correlated with the regulated genes (Figure 7B).

These findings underscore the importance of considering the unique structural features of fungal TPP riboswitches, particularly the variable P3 region, in detection and strategy. The development of fungal-specific models and careful consideration of P3 variability in sequence comparisons are crucial for the accurate identification and classification of TPP riboswitches in fungal genomes.

Further investigations are required to elucidate the underlying factors contributing to P3 variability and confirm the potential regulatory function of the putative TPPsw^SUGAR^ sequences. Understanding these structural nuances is essential to unravel the diverse roles of TPP riboswitches in fungal biology and their potential as targets for antifungal therapies.

### Genomic Context and Regulation Modes of TPP Riboswitches

The precise genomic position of the TPP riboswitch dictates its mode of regulation, consistent with previous findings reported in other studies.^61,65^ In this regard, our results corroborate previous observations from other studies.^61,65^ Within our data set, type I regulation (Figure S1) emerged as the predominant mode, with the majority of the TPP riboswitch sequences located in the 5′ UTR of the respective genes. Type III regulation (Figure S3) was the second most frequently observed mode, while Type II regulation (Figure S2) was only identified in a subset of the TPPsw^UREA^ sequences. Type IV regulation is relatively rare and found in a limited number of species, including *Malassezia furfur* and *Sporothrix schenckii*. We observed that TPPsw^SUGAR^ primarily employed type I regulation as its main mechanism, although type III regulation was also present. Consequently, most TPPsw^SUGAR^ sequences were positioned in the 5′-UTR of the corresponding genes.

## Discussion

Bioinformatics has a significant potential to drive breakthroughs in riboswitch research, particularly in identifying novel riboswitch groups. This approach allows for a comprehensive study of large genome data sets and the correlation of various aspects of riboswitch secondary structure conservation and genomic context, enhancing search robustness^88^.

Our analysis revealed limitations of previous search methods for TPP riboswitches in fungal organisms. For instance, we observed that several fungal TPP riboswitch sequences reported by Mukherjee et al.^65^ exhibited shorter lengths in the P3 region compared to our findings. This reduced length was often associated with apparently missing or poorly conserved 5′ portions of P1 and P2, as well as a portion of P3. These shorter sequences are potentially problematic as they may lack crucial structural elements of the complete riboswitch.

These observations led to incomplete identification of crucial structural elements in some riboswitch sequences. Specifically, Moldovan et al. previously argued that *Hortea werneckii* lacks TPP riboswitches that regulate thiamine biosynthesis genes.^61^ However, we identified both TPPsw^NMT1^ and TPPsw^THI4^ duplicates in the *H. werneckii* genome. Similarly, Mukherjee et al. did not detect certain TPPsw^AA^ sequences in Cryptococcus species.^65^

The size of the P3 stem plays a crucial role in detecting TPP riboswitches in fungal sequences, often constituting >60% of the total aptamer length. This extension significantly exceeded that observed in bacterial TPP riboswitches.^57,67^ Our results demonstrate that the P3 stem variability affects sequence alignment and clustering, highlighting the importance of accounting for P3 size when designing CMs for accurate detection of fungal TPP riboswitches.

The potential of TPP riboswitches as drug targets has been highlighted in previous studies.^92−94^ In vitro investigations using TPP analogs as antifungal agents have shown promise in controlling fungal growth, although there are limitations in their suitability as commercial drug candidates.^66,95^ Our comprehensive search identified TPP riboswitches in over 89% of the fungal species examined, with most genomes harboring two or more instances of these regulatory elements. This prevalence presents an exciting opportunity for antifungal drug development, potentially allowing the design of more potent therapeutic agents capable of simultaneously disrupting TPP production and availability in multiple instances.^66,95−99^

The discovery of a putative new group of TPP riboswitches associated with sugar transporters (TPPsw^SUGAR^) raises intriguing questions regarding the diversity of riboswitch functions in fungi. While this group shares key structural similarities with known TPP riboswitches, such as conserved nucleotides in the pyrimidine and pyrophosphate binding regions, it also presents distinctive characteristics.^100−107^ Notably, the TPPsw^SUGAR^ sequences exhibited variations in the P2, P3, and P4 stems as well as in the J2–4 region. While the P3 stem in TPPsw^SUGAR^ sequences is shorter on average compared to other fungal TPP riboswitch groups, it shows considerable sequence variability. This structural diversity, particularly in the P3 region, distinguishes TPPsw^SUGAR^ from other fungal TPP riboswitch groups, and may reflect adaptations to its specific regulatory role in sugar transport.

Genomic analysis of these TPPsw^SUGAR^ sequences revealed a consistent association with sugar transport genes in the 5′ UTR, primarily in Mucoromycota species, with some occurrences in Basidiomycota. This pattern suggests a potential regulatory role and lineage-specific adaptation of the TPPsw^SUGAR^. However, experimental validation is necessary to confirm their function as riboswitches and their specific roles in regulating sugar transporter genes. Further investigation of these structural variations could provide insight into the evolution and functional diversity of TPP riboswitches in fungi.

The prevalence of multiple TPP riboswitches within individual fungal genomes, particularly in species associated with aspergillosis or mucormycosis, may indicate that these pathogens could be more susceptible to therapeutic interventions targeting TPP riboswitches. The absence of TPP riboswitches in the human genome^57,67^ might offer an additional advantage, potentially resulting in fewer side effects and reduced harm to patients.

Our findings also highlight the need for further research into the diversity of fungal riboswitches. The differences in P3 stem length between bacteria and fungi, which hindered the detection of certain fungal organisms, suggest the possibility of undiscovered riboswitch classes in fungi that may possess substantial structural differences compared with bacterial riboswitches.

In summary, this study expands our understanding of TPP riboswitch diversity in fungi and emphasizes its potential as a promising target for antifungal therapies. The unique characteristics and prevalence of these riboswitches across diverse fungal species underscore their significance in fungal biology and in the development of novel therapeutic interventions. Future research should focus on experimental validation of the putative TPPsw^SUGAR^ group and exploration of the functional roles of multiple TPP riboswitches in individual fungal genomes.

## Conclusions

The discovery of a previously unknown group of putative TPP riboswitches in fungi that are associated with a family of sugar transporters presents exciting opportunities for drug targeting in fungal organisms. Our comprehensive analysis revealed that TPP riboswitches have the potential to interfere with various stages of the TPP transport and biosynthesis pathways. Remarkably, our investigation of a wide range of fungal species demonstrated that 89% of them possessed at least one TPP riboswitch, with the majority harboring two or more TPP riboswitches within their genomes. The TPPsw^THI4^ group exhibited the highest abundance in Ascomycota, whereas the TPPsw^AA^ group predominated in Basidiomycota, and TPPsw^SUGAR^ was primarily found in Mucoromycota.

Furthermore, our study highlights the impact of P3 size on the search for and alignment of fungal TPP riboswitches. To address the limitations of existing models, we developed novel covariance models tailored specifically for fungi, enabling the accurate identification of TPP riboswitch sequences. These findings not only expand our understanding of TPP riboswitch diversity in fungi but also emphasize their potential as promising targets for antifungal therapies. Although the identification of the putative TPPsw^SUGAR^ group is intriguing, we acknowledge the need for experimental validation to confirm its regulatory function and specificity for TPP. The unique characteristics and prevalence of these riboswitches across diverse fungal species underscore their significance in fungal biology and set the stage for future research on novel therapeutic interventions.

## Methods

### Data Collection

We retrieved 60 fungal TPP riboswitch sequences from the NCBI Nucleotide database based on the genome coordinates reported by Mukherjee et al.^65^ Sequences were equally distributed among the three fungal TPP riboswitch groups: NMT1,^108^ THI4,^109^ and urea transporters.^62^ We categorized sequences based on their associated target genes and used RNAfold to predict secondary structures. These were compared to the consensus structures reported by Mukherjee et al.^65^ using Varna^110^ for visualization. For sequences with shorter lengths in the P3 region, often associated with apparently missing or poorly conserved 5′ portions of P1 and P2 as well as part of P3, we extended the sequence by incorporating additional 5′ nucleotides until matching the consensus structure. This approach ensured that we captured the full length of the riboswitch, including all the crucial structural elements. We compiled a list of 131 medically relevant fungal species (Table S1) and obtained their complete genomes from the RefSeq database.^111^

### CM Construction

We downloaded the RF00059 covariance model (CM) from Rfam.^89−91^ Using the 60 refined sequences (20 each from NMT1, THI4, and urea transporter groups), we constructed three new CMs using Infernal’s cmbuild and cmcalibrate functions.^112−114^ These CMs served as comparison parameters to investigate the presence of riboswitches in medically relevant fungal genomes.

### TPP Riboswitch Search

We employed Infernal’s cmsearch to compare RF00059 CM and our three custom CMs against 131 fungal genomes. As the CM scores were not directly comparable, we selected the broadest genomic coordinates and manually refined them using the approach described for the initial 60 sequences. We validated secondary structures using RNAfold^115^ energy minimization and compared them to reported consensus structures, visualizing them with Varna.^110^ Sequence length was adjusted to match the consensus structures.

To identify open reading frames (ORFs) and verify the genomic context for each sequence, we used the NCBI ORFfinder and Artemis databases, respectively.^116,117^ To infer the gene product of the sequences and confirm annotation data, we conducted BLASTx searches against the UniProtKB database and the Transporter Classification Database (TCDB).^116,120,121^ Additionally, we detected conserved structural domains of putative transporters using DeepTMHMM.^122^

### Clustering of TPP Riboswitch Sequences and Tree Generation

The sequences were subjected to clustering using RNAclust, a Perl script that integrates tools from the Vienna RNA package, and LocARNA.^115,118^ RNAclust utilizes the base pair probability matrix of each sequence’s secondary structure distribution, calculated using RNAfold, to identify groups of RNA sequences sharing similar secondary structure motifs. The LocARNA tool generates a sequence-structure alignment for each pair of base-pair probability matrices. Hierarchical clustering (weighted pair group method with arithmetic mean) was then applied by RNAclust to construct a hierarchical cluster tree based on pairwise alignment distances. The optimal number of clusters is determined using the resulting tree. Finally, we used iTOL version 6 to visualize the cluster tree.^119^

### Alignment and Visualization of Secondary Structures

Alignments of TPP riboswitch sequences against the RF00059 CM were generated using the Infernal Cmalign tool.^113,114^ The resulting alignments were visualized using the Aline software, providing a comprehensive depiction of sequence conservation and variation.^123^ To further analyze the structural characteristics, consensus secondary structure models were generated using R2R software.^124^ These structural models provide an overview of the conserved folding patterns within TPP riboswitches, highlighting key elements crucial for their function and regulation.

## Supporting Information Available

Figures S1–S3 illustrating the generalized models of the types I, II, and III TPP riboswitch-based regulatory mechanisms in fungi (PDF)
Table S1 and supplementary file “SI - TPP riboswitches in medically relevant fungi.xlsx” providing details on the TPP riboswitches identified in fungal pathogens (XLSX)
Fasta file “SI_TPP_riboswitches.fasta” containing the sequences of the six groups of putative TPP riboswitches (TXT)

## References

[ref1] Rodrigues M. L.; Albuquerque P. C.; Reynolds T. B. Searching for a change: The need for increased support for public health and research on fungal diseases. PLoS Negl. Trop. Dis. 2018, 12 (6), e0006479 10.1371/journal.pntd.0006479. 29902170 PMC6001980

[ref2] Rodrigues M. L., Nosanchuk J. D. Fungal diseases as neglected pathogens: A wake-up call to public health officials. PLoS Negl. Trop. Dis. 2020, 14, (2), . 10.1371/journal.pntd.0007964 PMC7032689 32078635

[ref3] Infections GAF for F. GAFFI [Internet]. Available from: https://www.gaffi.org/antifungal-drug-maps/.

[ref4] Bhatt K.; Agolli A.; H. Patel M.; Garimella R.; Devi M.; Garcia E.; Amin H.; Domingue C.; Del Castillo R. G.; Sanchez-Gonzalez M.; et al. High mortality co-infections of COVID-19 patients: mucormycosis and other fungal infections. Discoveries. 2021, 9 (1), e126 10.15190/d.2021.5. 34036149 PMC8137279

[ref5] Basile K.; Halliday C.; Kok J.; Chen S. C. A. Fungal Infections Other Than Invasive Aspergillosis in COVID-19 Patients. J. Fungi. 2022, 8 (1), 58 10.3390/jof8010058. PMC8779574 35049999

[ref6] Fekkar A.; Lampros A.; Mayaux J.; Poignon C.; Demeret S.; Constantin J. M.; et al. Occurrence of invasive pulmonary fungal infections in patients with severe COVID-19 admitted to the ICU. Am. J. Respir Crit Care Med. 2021, 203 (3), 307–17. 10.1164/rccm.202009-3400OC. 33480831 PMC7874326

[ref7] Hoenigl M.; Seidel D.; Sprute R.; Cunha C.; Oliverio M.; Goldman G. H.; et al. COVID-19-associated fungal infections. Nature Microbiology. Nature Research 2022, 7, 1127–40. 10.1038/s41564-022-01172-2. PMC9362108 35918423

[ref8] Salehi M.; Khajavirad N.; Seifi A.; Salahshour F.; Jahanbin B.; Kazemizadeh H.; et al. Proven Aspergillus flavus pulmonary aspergillosis in a COVID- patient: A case report and review of the literature. Mycoses 2021, 809–816. 10.1111/myc.13255. 33576014 PMC8014135

[ref9] Rawson T. M.; Wilson R. C.; Holmes A. Understanding the role of bacterial and fungal infection in COVID-19. Clinical Microbiology and Infection [Internet]. 2021, 27 (1), 9–11. 10.1016/j.cmi.2020.09.025. 32979569 PMC7546203

[ref10] Villanueva-Lozano H.; Treviño-Rangel R de J; González G. M.; Ramírez-Elizondo M. T.; Lara-Medrano R.; Aleman-Bocanegra M. C.; et al. Outbreak of Candida auris infection in a COVID-19 hospital in Mexico. Clinical Microbiology and Infection. 2021, 27 (5), 813–6. 10.1016/j.cmi.2020.12.030. 33429028 PMC7835657

[ref11] Prestel C.; Anderson E.; Forsberg K.; Lyman M.; de Perio M. A.; Kuhar D.; et al. Candida auris Outbreak in a COVID-19 Specialty Care Unit — Florida, July–August 2020. MMWR Morb Mortal Wkly Rep. 2021, 70 (2), 56–7. 10.15585/mmwr.mm7002e3. 33444298 PMC7808709

[ref12] Hoenigl M.; Seidel D.; Carvalho A.; Rudramurthy S. M.; Arastehfar A.; Gangneux J. P.; et al. The emergence of COVID-19 associated mucormycosis: a review of cases from 18 countries. Lancet Microbe [Internet]. 2022, 3 (7), e543–52. 10.1016/S2666-5247(21)00237-8. 35098179 PMC8789240

[ref13] Barberis F.; Benedetti M. F.; de Abreu M. S.; Pola S. J.; Posse G.; Capece P.; Lausi A. F.; Nusblat A.; Cuestas M. L.; et al. Invasive fusariosis in a critically ill patient with severe COVID-19 pneumonia: A case report. Med. Mycol Case Rep. 2022, 35 (December 2021), 5–8. 10.1016/j.mmcr.2021.12.003. 34931158 PMC8673950

[ref14] Lahmer T.; Kriescher S.; Herner A.; Rothe K.; Spinner C. D.; Schneider J.; Mayer U.; Neuenhahn M.; Hoffmann D.; Geisler F.; Heim M.; Schneider G.; Schmid R. M.; Huber W.; Rasch S.; Magira E.; et al. Invasive pulmonary aspergillosis in critically ill patients with severe COVID-19 pneumonia: Results from the prospective AspCOVID-19 study. PLoS One [Internet]. 2021, 16 (3 March), e0238825–16. 10.1371/journal.pone.0238825. 33730058 PMC7968651

[ref15] Zurl C.; Hoenigl M.; Schulz E.; Hatzl S.; Gorkiewicz G.; Krause R.; Eller P.; Prattes J.; et al. Autopsy Proven Pulmonary Mucormycosis Due to Rhizopus microsporus in a Critically Ill COVID-19 Patient with Underlying Hematological Malignancy. J. Fungi. 2021, 7 (2), 88–4. 10.3390/jof7020088. PMC7912223 33513875

[ref16] Rahman F. I.; Islam M. R.; Bhuiyan M. A. Mucormycosis or black fungus infection is a new scare in South Asian countries during the COVID-19 pandemic: Associated risk factors and preventive measures. J. Med. Virol. 2021, 93 (12), 6447–8. 10.1002/jmv.27207. 34260073 PMC8426961

[ref17] Meshram H. S.; Kute V. B.; Chauhan S.; Desai S. Mucormycosis in post-COVID-19 renal transplant patients: A lethal complication in follow-up. Transplant Infect. Dis. 2021, 23 (4), 18–20. 10.1111/tid.13663. PMC8209936 34081817

[ref18] Borkar S. G.; Bajaj A. N. U. B. H. A. Mucormycosis: A Surge in Mucorales Fungal Infection in Post – Covid Patients in Indian States and Insight into Known and Unknown Factors. Int. J. Global Health. 2021, 01 (03), 26–60. 10.14302/issn.2693-1176.ijgh-21-3907.

[ref19] Trovato L.; Calvo M.; Migliorisi G.; Astuto M.; Oliveri F.; Oliveri S. Respiratory Medicine Case Reports Fatal VAP-related pulmonary aspergillosis by Aspergillus niger in a positive COVID-19 patient. Respiratory Medicine Case Reports 2021, 32 (January), 13–5. 10.1016/j.rmcr.2021.101367 PMC7890353 33619451

[ref20] Wiederhold N. P. Emerging Fungal Infections: New Species, New Names, and Antifungal Resistance. Clin Chem. 2021, 68 (1), 83–90. 10.1093/clinchem/hvab217. 34969112 PMC9383166

[ref21] Rudramurthy S. M.; Paul R. A.; Chakrabarti A. Invasive Aspergillosis by Aspergillus flavus: Epidemiology, Diagnosis, Antifungal. J. Fungi 2019, 55 10.3390/jof5030055. PMC6787648 31266196

[ref22] Hollingshead C.; Luttmann K.; Sitta E. A.; Elsaghir H. Aspergillus niger fungemia secondary to chronic pulmonary aspergillosis in a patient with invasive squamous cell carcinoma. BMJ Case Reports 2020, e234843 10.1136/bcr-2020-234843. PMC7078626 32188621

[ref23] Akimoto K.; Inoue H.; Jinno M.; Sato H.; Suganuma H.; Suzuki S.; et al. A Case of Bone Metastasis-Like Vertebral Aspergillosis Complicated with Pulmonary Aspergilloma After Lung Cancer Surgery. Am. J. Respir. Crit. Care Med. 2020, A5781–A5781. 10.1164/ajrccm-conference.2020.201.1.

[ref24] Altdorfer A.; Moerman F.; Weber T.; Maertens J.; Pirotte B. F. Respiratory Medicine Case Reports A rare case of invasive pulmonary aspergillosis presenting as organizing pneumonia due to Aspergillus niger in an immunocompetent host. Respir. Med. Case Rep 2021, 34 (August), 101503 10.1016/j.rmcr.2021.101503. 34485051 PMC8406023

[ref25] Spanamberg A.; Ravazzolo A. P.; Denardi L. B.; Hartz S. A.; Santurio J. M.; Driemeier D.; et al. Antifungal susceptibility profile of Aspergillus fumigatus isolates from avian lungs. Pesquisa Veterinaria Brasileira. 2020, 40 (2), 102–6. 10.1590/1678-5150-pvb-6297.

[ref26] Hope W. W.; McEntee L.; Livermore J.; Whalley S.; Johnson A.; Farrington N.; et al. Pharmacodynamics of the orotomides against Aspergillus fumigatus: New opportunities for treatment of multidrug-resistant fungal disease. mBio. 2017, 8 (4), 10-1128 10.1128/mbio.01157-17. PMC5565967 28830945

[ref27] Dietl A. M.; Meir Z.; Shadkchan Y.; Osherov N.; Haas H. Riboflavin and pantothenic acid biosynthesis are crucial for iron homeostasis and virulence in the pathogenic mold aspergillus fumigatus.. Virulence [Internet]. 2018, 9 (1), 1036–49. 10.1080/21505594.2018.1482181. 30052132 PMC6068542

[ref28] Friedman D. Z. P.; Schwartz I. S. Emerging fungal infections: New patients, new patterns, and new pathogens. J. Fungi. 2019, 5 (3), 67 10.3390/jof5030067. PMC6787706 31330862

[ref29] McCarthy M. W.; Walsh T. J. Drug development challenges and strategies to address emerging and resistant fungal pathogens.. Expert Rev. Anti Infect Ther [Internet]. 2017, 15 (6), 577–84. 10.1080/14787210.2017.1328279. 28480775

[ref30] Bouz G, Doležal M Advances in Antifungal Drug Development: An Up-To-Date Mini Review. Vol. 14, Pharmaceuticals. MDPI; 2021. 10.3390/ph14121312 PMC8706862 34959712

[ref31] Nivoix Y.; Ledoux M. P.; Herbrecht R. Antifungal Therapy: New and Evolving Therapies. Semin Respir Crit Care Med. 2020, 41 (1), 158–74. 10.1055/s-0039-3400291. 32000291

[ref32] Wall G, Lopez-Ribot J. L. Current antimycotics, new prospects, and future approaches to antifungal therapy. Vol. 9, Antibiotics. MDPI AG; 2020. p 1–10. 10.3390/antibiotics9080445 PMC7460292 32722455

[ref33] Perlin D. S.; Rautemaa-Richardson R.; Alastruey-Izquierdo A. The global problem of antifungal resistance: prevalence, mechanisms, and management. Lancet Infect Dis [Internet]. 2017, 17 (12), e383–92. 10.1016/S1473-3099(17)30316-X. 28774698

[ref34] Perlin D. S. Mechanisms of echinocandin antifungal drug resistance. Ann. N.Y. Acad. Sci. 2015, 1354 (1), 1–11. 10.1111/nyas.12831. 26190298 PMC4626328

[ref35] Shishodia S. K.; Tiwari S.; Shankar J. Resistance mechanism and proteins in Aspergillus species against antifungal agents.. Mycology [Internet]. 2019, 10 (3), 151–65. 10.1080/21501203.2019.1574927. 31448149 PMC6691784

[ref36] Vanden Bossche H.; Warnock D. W.; Dupont B.; Kerridge D.; Gupta S. S.; Improvisi L.; Marichal P.; Odds F. C.; Provost F.; Ronin O.; et al. Mechanisms and clinical impact of antifungal drug resistance. Med. Mycol. 1994, 32 (S1), 189–202. 10.1080/02681219480000821. 7722785

[ref37] Ghannoum M. A.; Rice L. B. Antifungal Agents: Mode of Action, Mechanisms of Resistance, and Correlation of These Mechanisms with Bacterial Resistance. Clin. Microbiol. Rev. 1999, 12 (4), 501–17. 10.1128/CMR.12.4.501. 10515900 PMC88922

[ref38] Wu Y.; Grossman N.; Totten M.; Memon W.; Fitzgerald A.; Ying C.; et al. Antifungal susceptibility profiles and drug resistance mechanisms of clinical lomentospora prolificans isolates. Antimicrob. Agents Chemother. 2020, 64 (11), 10-1128 10.1128/aac.00318-20. PMC7577128 32816726

[ref39] Kelly M.; Stevens R.; Konecny P. Lomentospora prolificans endocarditis - case report and literature review. BMC Infect Dis [Internet]. 2015, 16 (1), 1–5. 10.1186/s12879-016-1372-y. PMC4731902 26822980

[ref40] Kirchhoff L.; Dittmer S.; Weisner A. K.; Buer J.; Rath P. M.; Steinmann J. Antibiofilm activity of antifungal drugs, including the novel drug olorofim, against Lomentospora prolificans. J. Antimicrob. Chemother. 2020, 75 (8), 2133–40. 10.1093/jac/dkaa157. 32386411

[ref41] Pellon A.; Ramirez-Garcia A.; Buldain I.; Antoran A.; Martin-Souto L.; Rementeria A.; et al. Pathobiology of Lomentospora prolificans: could this species serve as a model of primary antifungal resistance?. Int. J. Antimicrob Agents [Internet]. 2018, 51 (1), 10–5. 10.1016/j.ijantimicag.2017.06.009. 28669833

[ref42] Jenks J. D.; Seidel D.; Cornely O. A.; Chen S.; van Hal S.; Kauffman C.; et al. Clinical characteristics and outcomes of invasive Lomentospora prolificans infections: Analysis of patients in the FungiScope® registry. Mycoses. 2020, 63 (5), 437–42. 10.1111/myc.13067. 32080902

[ref43] McManus D. S.; Shah S. Antifungal drugs. Side Effects of Drugs Annual. 2019, 41, 285–92. 10.1016/bs.seda.2019.09.002.

[ref44] Ito K. Inhaled antifungal therapy: benefits, challenges, and clinical applications.. Expert. Opin. Drug Delivery 2022, 19 (7), 755–15. 10.1080/17425247.2022.2084530. 35634895

[ref45] Scorzoni L.; de Paula e Silva A. C. A.; Marcos C. M.; Assato P. A.; de Melo W. C. M. A.; de Oliveira H. C.; Costa-Orlandi C. B.; Mendes-Giannini M. J. S.; Fusco-Almeida A. M.; et al. Antifungal therapy: New advances in the understanding and treatment of mycosis. Front Microbiol. 2017, 8 (JAN), 1–23. 10.3389/fmicb.2017.00036. 28167935 PMC5253656

[ref46] Gustafson C.; Koslosky M.; Leslie J.; Walczak C. Antifungal and Oral Anticancer Therapy Drug Interactions. Curr. Fungal Infect Rep. 2020, 14 (2), 130–40. 10.1007/s12281-020-00386-3.

[ref47] Francis P.; Walsh T. J. Evolving role of flucytosine in immunocompromised patients: New insights into safety, pharmacokinetics, and antifungal therapy. Clinical Infectious Diseases. 1992, 15 (6), 1003–18. 10.1093/clind/15.6.1003. 1457631

[ref48] Camargo J. F.; Jabr R.; Anderson A. D.; Lekakis L.; Diaz-Paez M.; Briski L. M.; et al. Successful Treatment of Disseminated Disease Due to Highly Resistant Aspergillus calidoustus with a Novel Antifungal Therapy. Antimicrob. Agents Chemother. 2022, 66 (3), e02206 10.1128/aac.02206-21. 34930032 PMC8923188

[ref49] Tverdek F. P.; Kofteridis D.; Kontoyiannis D. P. Antifungal agents and liver toxicity: a complex interaction. Expert Rev. Anti Infect Ther [Internet]. 2016, 14 (8), 765–76. 10.1080/14787210.2016.1199272. 27275514

[ref50] World Health Organization WHO fungal priority pathogens list to guide research, development and public health action [Internet]. 2022 [cited 2023 Jul 17]. Available from: https://www.who.int/publications/i/item/9789240060241.

[ref51] WHO GLOBAL PRIORITY LIST OF ANTIBIOTIC-RESISTANT BACTERIA TO GUIDE RESEARCH, DISCOVERY, AND DEVELOPMENT OF.

[ref52] Giarimoglou N, Kouvela A, Maniatis A, Papakyriakou A, Zhang J, Stamatopoulou V,A Riboswitch-Driven Era of New Antibacterials. Vol. 11, Antibiotics. MDPI; 2022. 10.3390/antibiotics11091243 PMC9495366 36140022

[ref53] Chen L.; Cressina E.; Dixon N.; Erixon K.; Agyei-Owusu K.; Micklefield J.; et al. Probing riboswitch-ligand interactions using thiamine pyrophosphate analogues. Org. Biomol Chem. 2012, 10 (30), 5924–31. 10.1039/c2ob07116a. 22514012

[ref54] Serganov A.; Polonskaia A.; Phan A. T.; Breaker R. R.; Patel D. J.; et al. Structural basis for gene regulation by a thiamine pyrophosphate-sensing riboswitch. Nature 2006, 441 (7097), 1167–71. 10.1038/nature04740. 16728979 PMC4689313

[ref55] Breaker R. R. Riboswitches and the RNA world. Cold Spring Harb Perspect Biol. 2012, 4 (2), a003566 10.1101/cshperspect.a003566. 21106649 PMC3281570

[ref56] Serganov A.; Nudler E. A Decade of Riboswitches. Cell 2013, 152 (0), 17–24. 10.1016/j.cell.2012.12.024. 23332744 PMC4215550

[ref57] Antunes D.; Jorge N. A. N.; Garcia de Souza Costa M.; Passetti F.; Caffarena E. R. Unraveling RNA dynamical behavior of TPP riboswitches: a comparison between Escherichia coli and Arabidopsis thaliana. Sci. Rep. 2019, 9 (1), 1–13. 10.1038/s41598-019-40875-1. 30862893 PMC6414600

[ref58] Ketzer P.; Kaufmann J. K.; Engelhardt S.; Bossow S.; Von Kalle C.; Hartig J. S.; et al. Artificial riboswitches for gene expression and replication control of DNA and RNA viruses. Proc. Natl. Acad. Sci. U. S. A. 2014, 111 (5), E554 10.1073/pnas.1318563111. 24449891 PMC3918795

[ref59] Regulski E. E.; Breaker R. R. In-line probing analysis of riboswitches. Methods Mol. Biol. 2008, 419 (5), 53–67. 10.1007/978-1-59745-033-1_4. 18369975

[ref60] Barrick J. E.; Breaker R. R. The distributions, mechanisms, and structures of metabolite-binding riboswitches. Genome Biol. 2007, 8 (11), R239 10.1186/gb-2007-8-11-r239. 17997835 PMC2258182

[ref61] Moldovan M. A.; Petrova S. A.; Gelfand M. S. Comparative genomic analysis of fungal TPP-riboswitches. Fungal Genet. Biol. [Internet]. 2018, 114 (February), 34–41. 10.1016/j.fgb.2018.03.004. 29548845

[ref62] Donovan P. D.; Holland L. M.; Lombardi L.; Coughlan A. Y.; Higgins D. G.; Wolfe K. H.; Butler G.; Mitchell A. P.; et al. TPP riboswitch-dependent regulation of an ancient thiamin transporter in Candida. PLoS Genet. 2018, 14 (5), e1007429–19. 10.1371/journal.pgen.1007429. 29852014 PMC5997356

[ref63] Vargas-Junior V.; Antunes D.; Guimarães A. C.; Caffarena E. In silico investigation of riboswitches in fungi: structural and dynamical insights into TPP riboswitches in Aspergillus oryzae.. RNA Biol. [Internet]. 2022, 19 (1), 90–103. 10.1080/15476286.2021.2015174. 34989318 PMC8786325

[ref64] Li S.; Breaker R. R. Eukaryotic TPP riboswitch regulation of alternative splicing involving long-distance base pairing. Nucleic Acids Res. 2013, 41 (5), 3022–31. 10.1093/nar/gkt057. 23376932 PMC3597705

[ref65] Mukherjee S.; Retwitzer M. D.; Barash D.; Sengupta S. Phylogenomic and comparative analysis of the distribution and regulatory patterns of TPP riboswitches in fungi. Sci. Rep. [Internet]. 2018, 8 (1), 1–13. 10.1038/s41598-018-23900-7. 29615754 PMC5882874

[ref66] Sudarsan N.; Cohen-Chalamish S.; Nakamura S.; Emilsson G. M.; Breaker R. R. Thiamine pyrophosphate riboswitches are targets for the antimicrobial compound pyrithiamine. Chem. Biol. 2005, 12 (12), 1325–35. 10.1016/j.chembiol.2005.10.007. 16356850

[ref67] Antunes D.; Jorge N. A. N.; Caffarena E. R.; Passetti F. Using RNA sequence and structure for the prediction of riboswitch aptamer: A comprehensive review of available software and tools. Front Genet. 2018, 8 (JAN), 1–16. 10.3389/fgene.2017.00231. PMC5780412 29403526

[ref68] Winkler W.; Nahvi A.; Breaker R. R. Thiamine derivatives bind messenger RNAs directly to regulate bacterial gene expression. Nature. 2002, 419 (6910), 952–6. 10.1038/nature01145. 12410317

[ref69] Eram M. S.; Ma K. Decarboxylation of pyruvate to acetaldehyde for ethanol production by hyperthermophiles. Biomolecules. 2013, 3 (3), 578–96. 10.3390/biom3030578. 24970182 PMC4030962

[ref70] Lonsdale D. A review of the biochemistry, metabolism and clinical benefits of thiamin(e) and its derivatives. Evidence-based Complementary and Alternative Medicine. 2006, 3 (1), 49–59. 10.1093/ecam/nek009. 16550223 PMC1375232

[ref71] Kawasaki T.; Sanemori H.; Egi Y.; Yoshida S.; Yamada K. Biochemical studies on pyrithiamine-resistant mutants of Escherichia coli K12. J. Biochem. 1976, 79 (5), 1035–42. 10.1093/oxfordjournals.jbchem.a131144. 783154

[ref72] Kubodera T.; Watanabe M.; Yoshiuchi K.; Yamashita N.; Nishimura A.; Nakai S.; et al. Thiamine-regulated gene expression of Aspergillus oryzae thiA requires splicing of the intron containing a riboswitch-like domain in the 5′-UTR. FEBS Lett. 2003, 555 (3), 516–20. 10.1016/S0014-5793(03)01335-8. 14675766

[ref73] De Jong L.; Meng Y.; Dent J.; Hekimi S. Thiamine pyrophosphate biosynthesis and transport in the nematode Caenorhabditis elegans. Genetics. 2004, 168 (2), 845–54. 10.1534/genetics.104.028605. 15514058 PMC1448845

[ref74] Mulhbacher J.; St-Pierre P.; Lafontaine D. A. Therapeutic applications of ribozymes and riboswitches. Curr. Opin Pharmacol. 2010, 10 (5), 551–6. 10.1016/j.coph.2010.07.002. 20685165

[ref75] Panchal V.; Brenk R. Riboswitches as drug targets for antibiotics. Antibiotics. 2021, 10 (1), 45–22. 10.3390/antibiotics10010045. 33466288 PMC7824784

[ref76] Maxwell C. S.; Sepulveda V. E.; Turissini D. A.; Goldman W. E.; Matute D. R. Recent admixture between species of the fungal pathogen Histoplasma. Evol Lett. 2018, 2 (3), 210–20. 10.1002/evl3.59. 30283677 PMC6121842

[ref77] Sanglard D. Emerging threats in antifungal-resistant fungal pathogens. Front Med. 2016, 3 (MAR), 11 10.3389/fmed.2016.00011. PMC4791369 27014694

[ref78] Forsberg K.; Woodworth K.; Walters M.; Berkow E. L.; Jackson B.; Chiller T.; et al. Candida auris: The recent emergence of a multidrug-resistant fungal pathogen. Med. Mycol. 2019, 57 (1), 1–12. 10.1093/mmy/myy054. 30085270

[ref79] da Glória Sousa M.; Reid D. M.; Schweighoffer E.; Tybulewicz V.; Ruland J.; Langhorne J.; Yamasaki S.; Taylor P. R.; Almeida S. R.; Brown G. D.; et al. Restoration of pattern recognition receptor costimulation to treat chromoblastomycosis, a chronic fungal infection of the skin. Cell Host Microb. 2011, 9 (5), 436–443. 10.1016/j.chom.2011.04.005. PMC3098964 21575914

[ref80] Arastehfar A.; Lass-Flörl C.; Garcia-Rubio R.; Daneshnia F.; Ilkit M.; Boekhout T.; Gabaldon T.; Perlin D. S.; et al. The quiet and underappreciated rise of drug-resistant invasive fungal pathogens. J. Fungi 2020, 6 (3), 138–34. 10.3390/jof6030138. PMC7557958 32824785

[ref81] Tsai C. K.; Liu Y. C.; Kuan A. S.; Lee K. L.; Yeh C. M.; Lee Y. T.; et al. Risk and impact of invasive fungal infections in patients with multiple myeloma. Ann. Hematol. 2020, 99 (8), 1813–22. 10.1007/s00277-020-04125-z. 32607596

[ref82] Turissini D. A.; Gomez O. M.; Teixeira M. M.; McEwen J. G.; Matute D. R. Species boundaries in the human pathogen Paracoccidioides. Fungal Genet. Biol. 2017, 106 (January), 9–25. 10.1016/j.fgb.2017.05.007. 28602831 PMC8335726

[ref83] Lockhart S. R.; Guarner J. Emerging and reemerging fungal infections.. Semin Diagn Pathol [Internet]. 2019, 36 (3), 177–81. 10.1053/j.semdp.2019.04.010. 31010605 PMC11979780

[ref84] Vandeputte P.; Ferrari S.; Coste A. T. Antifungal resistance and new strategies to control fungal infections. Int. J. Microbiol. 2012, 1 10.1155/2012/713687. 22187560 PMC3236459

[ref85] Lenassi M.; Gostinčar C.; Jackman S.; Turk M.; Sadowski I.; Nislow C.; et al. Whole Genome Duplication and Enrichment of Metal Cation Transporters Revealed by De Novo Genome Sequencing of Extremely Halotolerant Black Yeast Hortaea werneckii. PLoS One 2013, 8 (8), e71328 10.1371/journal.pone.0071328. 23977017 PMC3744574

[ref86] Romeo O.; Marchetta A.; Giosa D.; Giuffrè L.; Urzì C.; De Leo F. Whole genome sequencing and comparative genome analysis of the halotolerant deep sea black yeast hortaea werneckii. Life 2020, 10 (10), 229 10.3390/life10100229. 33023088 PMC7601665

[ref87] Sinha S.; Flibotte S.; Neira M.; Formby S.; Plemenitas A.; Cimerman N. G.; et al. Insight into the recent genome duplication of the halophilic yeast Hortaea werneckii: Combining an improved genome with gene expression and chromatin structure. G3: Genes, Genomes. Genetics. 2017, 7 (7), 2015–22. 10.1534/g3.117.040691. PMC5499112 28500048

[ref88] Kavita K, Breaker R. R. Discovering riboswitches: the past and the future. Trends in Biochemical Sciences; Elsevier Ltd; 2022. 10.1016/j.tibs.2022.08.009 PMC10043782 36150954

[ref89] Griffiths-Jones S.; Bateman A.; Marshall M.; Khanna A.; Eddy S. R. Rfam: An RNA family database. Nucleic Acids Res. 2003, 31 (1), 439–41. 10.1093/nar/gkg006. 12520045 PMC165453

[ref90] Kalvari I.; Nawrocki E. P.; Argasinska J.; Quinones-Olvera N.; Finn R. D.; Bateman A.; Petrov A. I.; et al. Non-Coding RNA Analysis Using the Rfam Database. Curr. Protoc Bioinformatics. 2018, 62 (1), 1–44. 10.1002/cpbi.51. PMC6754622 29927072

[ref91] Kalvari I.; Argasinska J.; Quinones-Olvera N.; Nawrocki E. P.; Rivas E.; Eddy S. R.; et al. Rfam 13.0: Shifting to a genome-centric resource for non-coding RNA families. Nucleic Acids Res. 2018, 46 (D1), D335–42. 10.1093/nar/gkx1038. 29112718 PMC5753348

[ref92] Traykovska M.; Otcheva L. A.; Penchovsky R. Targeting TPP Riboswitches Using Chimeric Antisense Oligonucleotide Technology for Antibacterial Drug Development. ACS Appl. Bio Mater. 2022, 5 (10), 4896–902. 10.1021/acsabm.2c00628. 36170638

[ref93] Blount K. F.; Breaker R. R. Riboswitches as antibacterial drug targets. Nat. Biotechnol. 2006, 24, 1558–64. 10.1038/nbt1268. 17160062

[ref94] Lünse C. E.; Schüller A.; Mayer G. The promise of riboswitches as potential antibacterial drug targets. International Journal of Medical Microbiology. 2014, 304, 79–92. 10.1016/j.ijmm.2013.09.002. 24140145

[ref95] Thore S.; Frick C.; Ban N. Structural basis of thiamine pyrophosphate analogues binding to the eukaryotic riboswitch. J. Am. Chem. Soc. 2008, 130 (26), 8116–7. 10.1021/ja801708e. 18533652

[ref96] Bongomin F.; Kwizera R.; Denning D. W. Getting histoplasmosis on the map of international recommendations for patients with advanced hiv disease. J. Fungi 2019, 5 (3), 80 10.3390/jof5030080. PMC6787619 31480775

[ref97] Morote S.; Nacher M.; Blaizot R.; Ntab B.; Blanchet D.; Drak Alsibai K.; Demar M.; Djossou F.; Couppié P.; Adenis A.; et al. Comparison of disseminated histoplasmosis with and without cutaneo-mucous lesions in persons living with hiv in French Guiana. J. Fungi 2020, 6 (3), 133 10.3390/jof6030133. PMC7557946 32806526

[ref98] Suleyman G.; Alangaden G. J. Nosocomial Fungal Infections: Epidemiology, Infection Control, and Prevention. Infect Dis Clin North Am. 2021, 35 (4), 1027–53. 10.1016/j.idc.2021.08.002. 34752219

[ref99] Ruhnke M.; Behre G.; Buchheidt D.; Christopeit M.; Hamprecht A.; Heinz W.; et al. Diagnosis of invasive fungal diseases in haematology and oncology: 2018 update of the recommendations of the infectious diseases working party of the German society for hematology and medical oncology (AGIHO). Mycoses. 2018, 61 (11), 796–813. 10.1111/myc.12838. 30098069

[ref100] Gralak M. A.; Dębski B.; Drywień M. Thiamine deficiency affects glucose transport and β-oxidation in rats. J. Anim Physiol Anim Nutr (Berl). 2019, 103 (5), 1629–35. 10.1111/jpn.13146. 31259440 PMC6851678

[ref101] Larkin J. R.; Zhang F.; Godfrey L.; Molostvov G.; Zehnder D.; Rabbani N.; et al. Glucose-Induced Down Regulation of Thiamine Transporters in the Kidney Proximal Tubular Epithelium Produces Thiamine Insufficiency in Diabetes. PLoS One. 2012, 7 (12), e53175 10.1371/journal.pone.0053175. 23285265 PMC3532206

[ref102] Liang X.; Chien H. C.; Yee S. W.; Giacomini M. M.; Chen E. C.; Piao M.; et al. Metformin Is a Substrate and Inhibitor of the Human Thiamine Transporter, THTR-2 (SLC19A3). Mol. Pharmaceutics 2015, 12 (12), 4301–10. 10.1021/acs.molpharmaceut.5b00501. PMC4800991 26528626

[ref103] Peng M.; Aguilar-Pontes M. V.; de Vries R. P.; Mäkelä M. R. In silico analysis of putative sugar transporter genes in Aspergillus niger using phylogeny and comparative transcriptomics. Front Microbiol. 2018, 9 (MAY), 1–10. 10.3389/fmicb.2018.01045. 29867914 PMC5968117

[ref104] Nogueira K. M. V.; de Paula R. G.; Antoniêto A. C. C.; dos Reis T. F.; Carraro C. B.; Silva A. C.; Almeida F.; Rechia C. G. V.; Goldman G. H.; Silva R. N.; et al. Characterization of a novel sugar transporter involved in sugarcane bagasse degradation in Trichoderma reesei. Biotechnol Biofuels. 2018, 11 (1), 1–17. 10.1186/s13068-018-1084-1. 29619080 PMC5879799

[ref105] Mäkelä M. R.; Aguilar-Pontes M. V.; Van Rossen-Uffink D.; Peng M.; De Vries R. P. The fungus Aspergillus Niger consumes sugars in a sequential manner that is not mediated by the carbon catabolite repressor CreA. Sci. Rep. 2018, 8 (1), 6655 10.1038/s41598-018-25152-x. 29703914 PMC5923239

[ref106] Podolsky I. A.; Seppälä S.; Xu H.; Jin Y. S.; O'Malley M. A. A SWEET surprise: Anaerobic fungal sugar transporters and chimeras enhance sugar uptake in yeast. Metab Eng. 2021, 66 (September 2020), 137–47. 10.1016/j.ymben.2021.04.009. 33887459

[ref107] Mukherjee P. K.; Chandra J.; Yu C.; Sun Y.; Pearlman E.; Ghannoum M. A. Characterization of Fusarium Keratitis Outbreak Isolates: Contribution of Biofilms to Antimicrobial Resistance and Pathogenesis. Invest Ophthalmol Vis Sci. 2012, 53 (8), 4450–7. 10.1167/iovs.12-9848. 22669723 PMC3394686

[ref108] Maundrell K. nmt1 of fission yeast. A highly transcribed gene completely repressed by thiamine. J. Biol. Chem. 1990, 265 (19), 10857–64. 10.1016/S0021-9258(19)38525-4. 2358444

[ref109] Chatterjee A.; Jurgenson C. T.; Schroeder F. C.; Ealick S. E.; Begley T. P. Biosynthesis of thiamin thiazole in eukaryotes: Conversion of NAD to an advanced intermediate. J. Am. Chem. Soc. 2007, 129 (10), 2914–22. 10.1021/ja067606t. 17309261 PMC2536526

[ref110] Darty K.; Denise A.; Ponty Y. VARNA: Interactive drawing and editing of the RNA secondary structures. Bioinf. App. Note 2009, 25 (15), 1974 10.1093/bioinformatics/btp250. PMC2712331 19398448

[ref111] O’Leary N. A.; Wright M. W.; Brister J. R.; Ciufo S.; Haddad D.; McVeigh R.; et al. Reference sequence (RefSeq) database at NCBI: Current status, taxonomic expansion, and functional annotation. Nucleic Acids Res. 2016, 44 (D1), D733–45. 10.1093/nar/gkv1189. 26553804 PMC4702849

[ref112] Eddy S. R.; Durbin R. RNA sequence analysis using covariance models. Nucleic Acids Res. 1994, 22 (11), 2079–88. 10.1093/nar/22.11.2079. 8029015 PMC308124

[ref113] Nawrocki E. P. Annotating Functional RNAs in Genomes Using Infernal. Methods Mol. Biol. 2014, 1097, 163–197. 10.1007/978-1-62703-709-9_9. 24639160

[ref114] Nawrocki E. P.; Eddy S. R. Infernal 1.1:100-fold faster RNA homology searches. Bioinformatics. 2013, 29 (22), 2933–5. 10.1093/bioinformatics/btt509. 24008419 PMC3810854

[ref115] Lorenz R.; Bernhart S. H.; Höner zu Siederdissen C.; Tafer H.; Flamm C.; Stadler P. F.; Hofacker I. L. ViennaRNA Package 2.0. Algorithms for Molecular Biology [Internet]. 2011, 6, 1–26. 10.1186/1748-7188-6-26. 22115189 PMC3319429

[ref116] Sayers E. W.; Bolton E. E.; Brister J. R.; Canese K.; Chan J.; Comeau D. C.; et al. Database resources of the national center for biotechnology information. Nucleic Acids Res. 2022, 50 (D1), D20–6. 10.1093/nar/gkab1112. 34850941 PMC8728269

[ref117] Carver T.; Harris S. R.; Berriman M.; Parkhill J.; McQuillan J. A. Artemis: An integrated platform for visualization and analysis of high-throughput sequence-based experimental data. Bioinformatics. 2012, 28 (4), 464–9. 10.1093/bioinformatics/btr703. 22199388 PMC3278759

[ref118] Will S.; Joshi T.; Hofacker I. L.; Stadler P. F.; Backofen R. LocARNA-P: Accurate boundary prediction and improved detection of structural RNAs. Rna. 2012, 18 (5), 900–14. 10.1261/rna.029041.111. 22450757 PMC3334699

[ref119] Letunic I.; Bork P. Interactive tree of life (iTOL) v5: An online tool for phylogenetic tree display and annotation. Nucleic Acids Res. 2021, 49 (W1), W293–6. 10.1093/nar/gkab301. 33885785 PMC8265157

[ref120] UniProt: The universal protein knowledgebase. Nucleic Acids Res. 2017, 45, (D1), , D158, 10.1093/nar/gkw1099. 27899622 PMC5210571

[ref121] Saier M. H.; Reddy V. S.; Moreno-Hagelsieb G.; Hendargo K. J.; Zhang Y.; Iddamsetty V.; et al. The transporter classification database (TCDB): 2021 update. Nucleic Acids Res. 2021, 49 (D1), D461–7. 10.1093/nar/gkaa1004. 33170213 PMC7778945

[ref122] Hallgren J., Tsirigos K. D., Damgaard Pedersen M., Juan J., Armenteros A., Marcatili P.,DeepTMHMM predicts alpha and beta transmembrane proteins using deep neural networks. 10.1101/2022.04.08.487609.

[ref123] Bond C. S.; Schüttelkopf A. W. ALINE: A WYSIWYG protein-sequence alignment editor for publication-quality alignments. Acta Crystallogr. D Biol. Crystallogr. 2009, 65 (5), 510–2. 10.1107/S0907444909007835. 19390156

[ref124] Weinberg Z. R2R–software to speed the depiction of aesthetic consensus RNA secondary structures. BMC Bioinf. 2016, 12, 3 10.1186/1471-2105-12-3. PMC3023696 21205310

