# Supplementary material for: Genome-Wide Exploration of Thiamin Pyrophosphate Riboswitches in Medically Relevant Fungi Reveals Diverse Distribution and Implications for Antimicrobial Drug Targeting
Source: ACS Omega. 2024 Dec 10;9(51):50134–46. doi: 10.1021/acsomega.4c00158 (PMC11683625; doi:10.1021/acsomega.4c00158)
Supplement: Supplementary file 1 — ao4c00158_si_001.pdf [file ao4c00158_si_001.pdf]

Supporting information for

Genome-Wide Exploration of TPP Riboswitches in Medically Relevant Fungi Reveals Diverse Distribution and Implications for Antimicrobial Drug Targeting

Valdemir Vargas-Junior[1], Ana Carolina Ramos Guimarães[1], Ernesto Raul Caffarena[2] and Deborah Antunes[1*]

[1]Laboratory for Applied Genomics and Bioinnovations, Oswaldo Cruz Institute (IOC - FIOCRUZ), Rio de Janeiro, Brazil
[2]Computational Biophysics and Molecular Modeling Group, Scientific Computing Program (PROCC - FIOCRUZ), Rio de Janeiro, Brazil
(*) Corresponding Author. E-mail address: deborah.santos@fiocruz.br (D. Antunes)

Contents:

Figure S1. A generalized model of the Type I TPP riboswitch-based regulation mechanism in fungi...............2

Figure S2. A generalized model of the Type II TPP riboswitch-based regulation mechanism in fungi. 3

Figure S3. A generalized model of the Type III TPP riboswitch-based regulation mechanism in fungi. 4

Table S1. TPP riboswitches identified in fungal pathogens...5

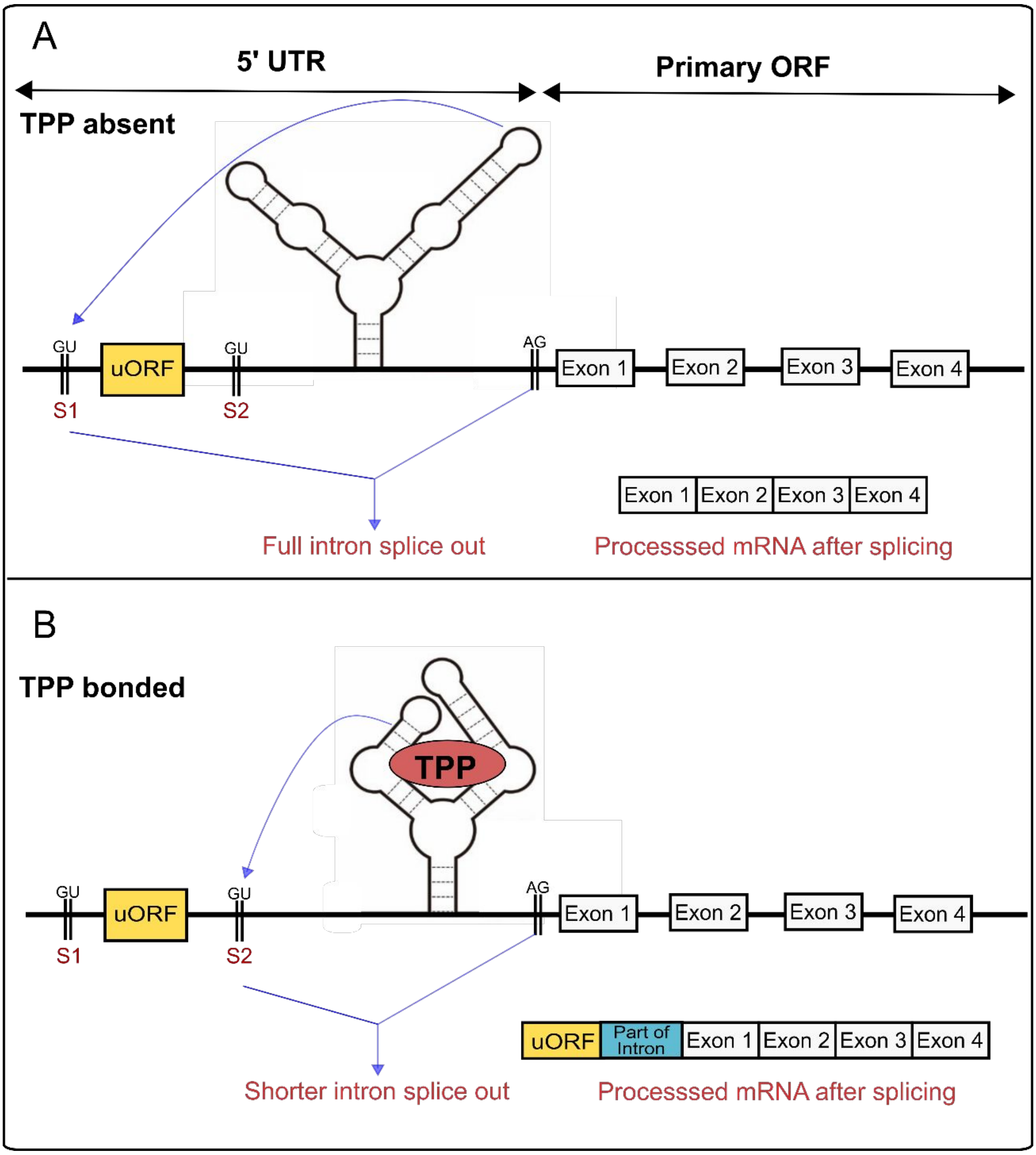

Figure S1. A generalized model of the Type I TPP riboswitch-based regulation mechanism in fungi. This mechanism is observed when the riboswitch is located in the intron of the 5′ UTR of the corresponding genes in fungi. **A)** TPP-free state of the riboswitch, which promotes the interaction with the S1 site and splicing of the full intron containing a short uORF. **B)** TPP-bound state of the riboswitch, which promotes the interaction with the S2 site and splicing of the shorter intron. The thin black line between successive exons represents the introns. The uORF is highlighted in yellow. The arrow pointing towards GU indicates activation of the corresponding splice site. The resulting processed mRNA obtained after splicing is shown at the bottom of the figure.

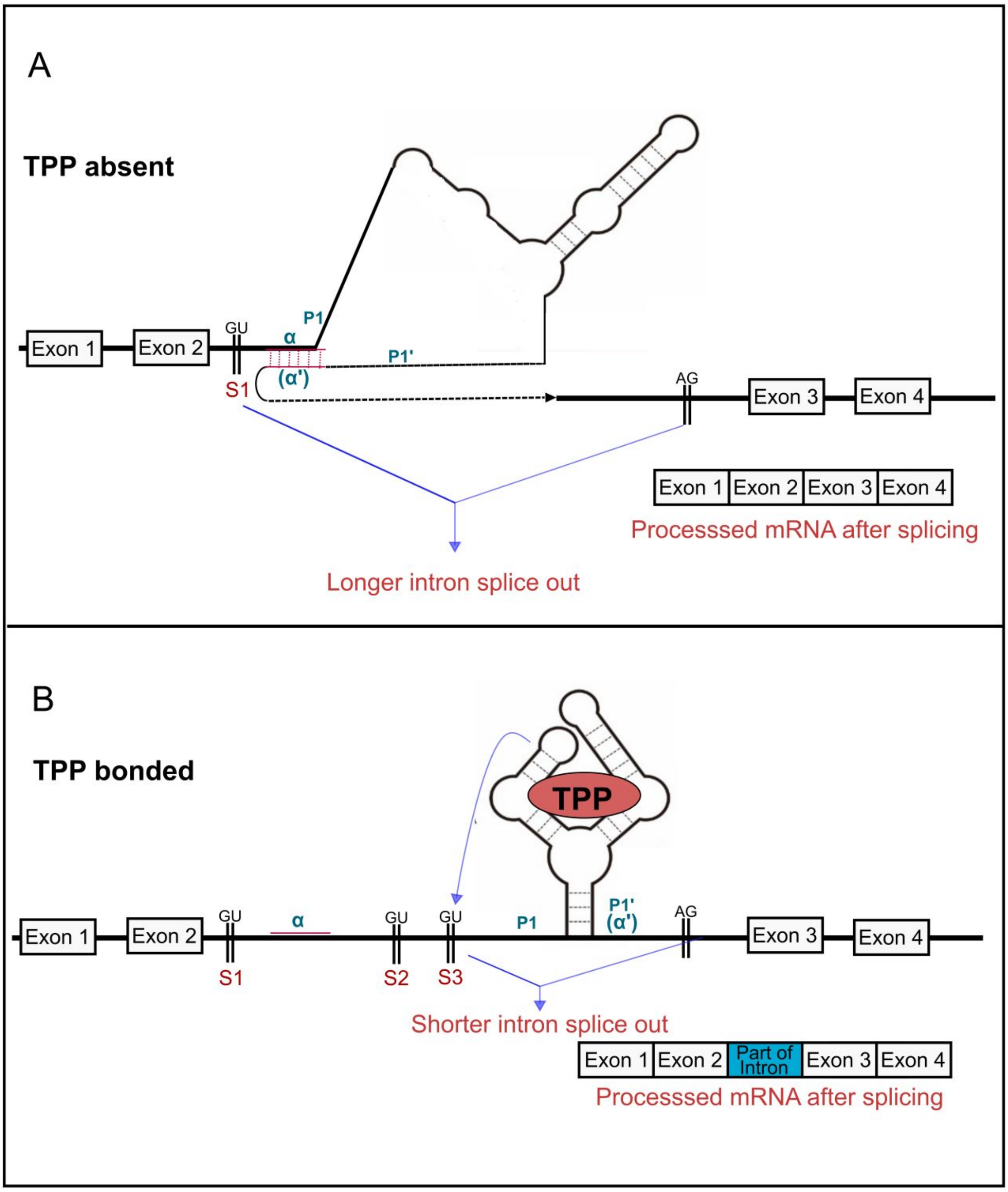

Figure S2. A generalized model of the Type II TPP riboswitch-based regulation mechanism in fungi. This mechanism is observed when the riboswitch is present in the large internal intron of the transporter genes in fungi and involved in long-distance base-pairing. **A)** TPP-free state of the riboswitch which promotes the long-distance base pairing of the P1′ (α′) stem of the TPP aptamer with a distal α segment near the S1 site. Such interaction promotes the splicing of the larger intron. **B)** TPP-bound state of the riboswitch where the P1′ (α′) segment is incorporated into the aptamer, forming a stable pairing with a complementary segment in the P1 stem. Such conformational changes promote interaction with alternative splice sites and splicing of the shorter intron portion. The thin black line between successive exons represents the introns. The arrow pointing towards GU indicates activation of the corresponding splice site. The resulting processed mRNA obtained after splicing is shown at the bottom of the figure.

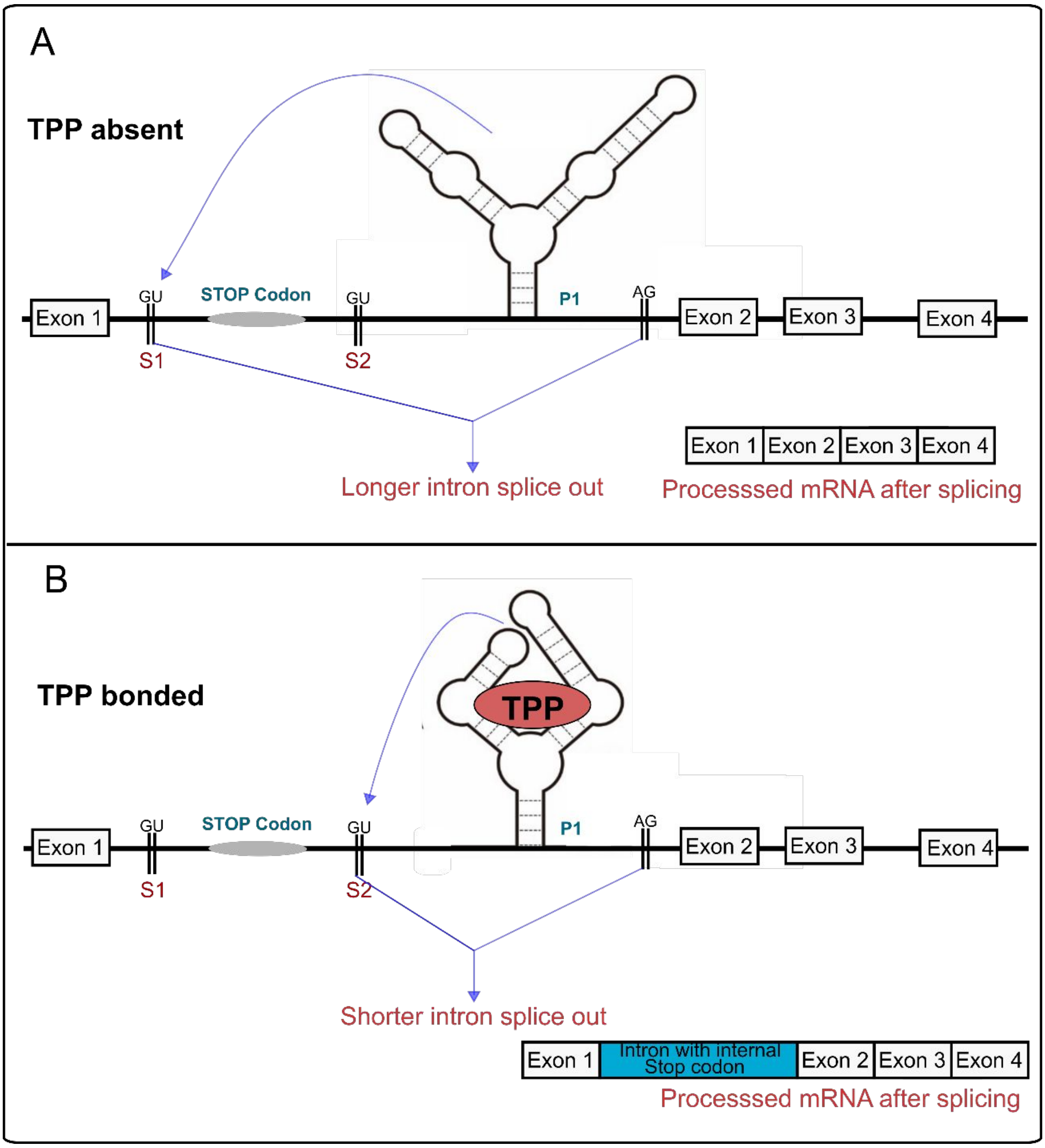

Figure S3. A generalized model of the Type III TPP riboswitch-based regulation mechanism in fungi. This mechanism is observed when the riboswitch is in any intermediate intron of the corresponding biosynthesis/transporter genes in fungi. **A)** TPP-free state of the riboswitch, which promotes the interaction with the S1 site and splicing of the full intron containing the internal stop codon. **B)** TPP-bound state of the riboswitch, which promotes the interaction with the S2 site and splicing of the shorter intron. The thin black line between successive exons represents the introns. The arrow pointing towards GU indicates activation of the corresponding splice site. In the TPP-bound state, the internal stop codon ensures premature translation termination, resulting in a dysfunctional protein product.

Table S1. TPP riboswitches identified in fungal pathogens.

SPECIE	SEQUENCE ID	START	END	STRAND	GENE PRODUCT	LOCATION	REGULATION TYPE
Alternaria alternata	NW_017306194.1	267509	267636	+	NMT1	5' UTR	Type I
Alternaria alternata	NW_017306206.1	376334	376240	-	NUCLEOSIDE TRANSPORTER	INTRON	Tiype III
Alternaria alternata	NW_017306202.1	357429	357280	-	THI4	5' UTR	Type I
Alternaria alternata	NW_017306190.1	96081	95940	-	UREA	INTRON	Type II
Aspergillus flavus	NC_054694.1	3452553	3452780	+	NMT1	5' UTR	Type I
Aspergillus flavus	NC_054692.1	2310868	2311038	+	THI4	5' UTR	Type I
Aspergillus flavus	NC_054693.1	3294596	3294414	-	NUCLEOSIDE TRANSPORTER	5' UTR	Type I
Aspergillus fumigatus	NC_007198.1	638474	638271	-	NMT1	5' UTR	Type I
Aspergillus fumigatus	NC_007199.1	1957935	1958107	+	THI4	5' UTR	Type I
Aspergillus fumigatus	NC_007195.1	768658	768836	+	NUCLEOSIDE TRANSPORTER	5' UTR	Type I
Aspergillus latus	VCRL01000207.1	37584	37492	-	NMT1	5' UTR	Type I
Aspergillus latus	VCRL01000038.1	451277	451369	+	NMT1	5' UTR	Type I
Aspergillus latus	VCRL01000015.1	589916	590071	+	NUCLEOSIDE TRANSPORTER	5' UTR	Type I
Aspergillus latus	VCRL01000007.1	285516	285669	+	THI4	5' UTR	Type I
Aspergillus latus	VCRL01000003.1	189472	189345	-	NUCLEOSIDE TRANSPORTER	5' UTR	Type I
Aspergillus latus	VCRL01000002.1	609870	609999	+	THI4	5' UTR	Type I
Aspergillus lentulus	NW_022983924.1	11634	11724	+	AMINO ACID TRANSPORTER	5' UTR	Type I
Aspergillus lentulus	NW_022983871.1	222472	222678	+	NMT1	5' UTR	Type I
Aspergillus lentulus	NW_022983873.1	90787	90616	-	THI4	5' UTR	Type I
Aspergillus lentulus	NW_022983890.1	228561	228740	+	NUCLEOSIDE TRANSPORTER	5' UTR	Type I
Aspergillus fischeri	NW_001509772.1	2148287	2148486	+	NMT1	5' UTR	Type I
Aspergillus fischeri	NW_001509760.1	2084762	2084933	+	THI4	5' UTR	Type I
Aspergillus fischeri	NW_001509772.1	2148288	2148485	+	NUCLEOSIDE TRANSPORTER	5' UTR	Type I
Aspergillus oryzae	NC_036438.1	3550250	3550477	+	NMT1	INTRON	Type III
Aspergillus oryzae	NC_036436.1	2327557	2327727	+	THI4	INTRON	Type III
Aspergillus oryzae	NC_036437.1	3333867	3333685	-	NUCLEOSIDE TRANSPORTER	5' UTR	Type I
Aspergillus nidulans	NT_107005.1	483184	483092	-	NMT1	5' UTR	Type I
Aspergillus nidulans	NT_107012.1	1442020	1441867	-	THI4	5' UTR	Type I
Aspergillus nidulans	NT_107011.1	735089	734964	-	NUCLEOSIDE TRANSPORTER	5' UTR	Type I
Aspergillus niger	NT_166526.1	398427	398559	+	THI4	INTRON	Type III
Aspergillus niger	NT_166519.1	2566917	2567037	+	NMT1	INTRON	Type III
Aspergillus niger	NT_166523.1	398426	398560	+	NUCLEOSIDE TRANSPORTER	5' UTR	Type I
Aspergillus terreus	NT_165937.1	574748	574644	-	NMT1	5' UTR	Type I
Aspergillus terreus	NT_165931.1	509334	509212	-	THI4	5' UTR	Type I
Aspergillus terreus	NT_165938.1	314869	314776	-	NUCLEOSIDE TRANSPORTER	5' UTR	Type I

Aureobasidium pullulans	NW_021941022.1	1576480	1576382	-	NUCLEOSIDE TRANSPORTER	INTRON	Type III
Aureobasidium pullulans	NW_021941036.1	411447	411599	+	THI4	5' UTR	Type I
Aureobasidium pullulans	NW_021941056.1	11417	11478	+	NMT1	5' UTR	Type I
Aureobasidium pullulans	NW_021941030.1	740501	740393	-	AMINO ACID TRANSPORTER	INTRON	Type III
Beauveria bassiana	NW_007930843.1	983369	983237	-	NMT1	5' UTR	Type I
Beauveria bassiana	NW_007930847.1	234797	234650	-	THI4	5' UTR	Type I
Beauveria bassiana	NW_007930862.1	223697	223553	-	UREA TRANSPORTER	INTRON	Type II
Blastomyces dermatitidis	EQ999981.1	2341828	2342052	+	NMT1	INTRON	Type III
Blastomyces dermatitidis	EQ999981.1	1797093	1796912	-	THI4	INTRON	Type III
Blastomyces dermatitidis	EQ999978.1	1531492	1531675	+	UREA TRANSPORTER	INTRON	Type II
Candida auris	NW_021640167.1	410887	410801	-	UREA TRANSPORTER	INTRON	Type II
Candida haemulonis	NW_020289912.1	594417	594311	-	UREA TRANSPORTER	INTRON	Type II
Candida inconspicua	SELW01000121.1	185329	185262	-	UREA TRANSPORTER	INTRON	Type II
Candida orthopsilosis	NC_018295.1	470511	470414	-	UREA TRANSPORTER	INTRON	Type II
Candida parapsilosis	NW_023503282.1	474597	474499	-	UREA TRANSPORTER	INTRON	Type II
Cladophialophora bantiana	NW_015971594.1	333146	332991	-	UREA TRANSPORTER	5' UTR	Type I
Cladophialophora bantiana	NW_015971583.1	637324	637153	-	THI4	INTRON	Type III
Cladophialophora carrionii	NW_008481828.1	1236287	1236125	-	THI4	INTRON	Type III
Cladophialophora carrionii	NW_008481818.1	437462	437314	-	UREA TRANSPORTER	INTRON	Type II
Cladosporium cladosporioides	NOXB01000001.1	1465021	1464872	-	NUCLEOSIDE TRANSPORTER	INTRON	Type III
Cladosporium cladosporioides	NOXB01000005.1	1647981	1647751	-	THI4	INTRON	Type III
Cladosporium cladosporioides	NOXB01000011.1	129596	129423	-	NMT1	INTRON	Type III
Cladosporium cladosporioides	NOXB01000007.1	1089978	1090144	+	AMINO ACID TRANSPORTER	5' UTR	Type I
Cladosporium sphaerospermum	JAHARS010000006.1	734718	734574	-	NUCLEOSIDE TRANSPORTER	INTRON	Type III
Cladosporium sphaerospermum	JAHARS010000025.1	175308	175075	-	THI4	INTRON	Type III
Cladosporium sphaerospermum	JAHARS010000008.1	101187	101008	-	NMT1	INTRON	Type III
Coccidioides immitis	NW_004504309.1	435807	435584	-	NMT1	INTRON	Type III
Coccidioides immitis	NW_004504310.1	5557045	5557239	+	THI4	INTRON	Type III
Coccidioides immitis	NW_004504310.1	7346905	7347069	+	UREA TRANSPORTER	INTRON	Type II
Coccidioides posadasii	NW_003316003.1	290607	290383	-	NMT1	5' UTR	Type I
Coccidioides posadasii	NW_003315979.1	4347745	4347909	+	UREA TRANSPORTER	INTRON	Type II
Coccidioides posadasii	NW_003315979.1	2614842	2615037	+	THI4	5' UTR	Type I
Colletotrichum coccodes	LECQ01000186.1	36510	36646	+	THI4	5' UTR	Type I

Colletotrichum coccodes	LECQ01000205.1	56397	56231	-	NMT1	5' UTR	Type I
Colletotrichum coccodes	LECQ01000202.1	74511	74364	-	UREA TRANSPORTER	INTRON	Type II
Colletotrichum coccodes	LECQ01000187.1	5646	5810	+	NMT1	5' UTR	Type I
Coniochaeta hoffmannii	NXFW01000056.1	89043	89209	+	NMT1	5' UTR	Type I
Coniochaeta hoffmannii	NXFW01000004.1	79135	79291	+	THI4	5' UTR	Type I
Coniochaeta hoffmannii	NXFW01000003.1	234903	235053	+	UREA TRANSPORTER	INTRON	Type II
Curvularia lunata	PELC01000050.1	171242	171151	-	NUCLEOSIDE TRANSPORTER	5' UTR	Type I
Curvularia lunata	PELC01000052.1	75236	75384	+	THI4	5' UTR	Type I
Curvularia lunata	PELC01000085.1	80784	80903	+	NMT1	5' UTR	Type I
Curvularia lunata	PELC01000038.1	153417	153549	+	UREA TRANSPORTER	INTRON	Type II
Debaryomyces hansenii	NC_006047.2	1846432	1846556	+	UREA TRANSPORTER	INTRON	Type III
Epicoccum nigrum	KZ107839.1	3124268	3124417	+	THI4	5' UTR	Type I
Epicoccum nigrum	KZ107841.1	555190	555039	-	NMT1	5' UTR	Type I
Epicoccum nigrum	KZ107870.1	7181	7061	-	UREA TRANSPORTER	INTRON	Type II
Epicoccum nigrum	KZ107845.1	513277	513432	+	NUCLEOSIDE TRANSPORTER	5' UTR	Type I
Epicoccum nigrum	KZ107839.1	1731181	1731349	+	NUCLEOSIDE TRANSPORTER	5' UTR	Type I
Emergomyces africanus	LGUA01000051.1	14941	14769	-	THI4	5' UTR	Type I
Emergomyces africanus	LGUA01001479.1	3695	3897	+	NMT1	5' UTR	Type I
Emergomyces africanus	LGUA01000225.1	11132	11308	+	UREA	5' UTR	Type I
Emergomyces pasteurianus	LGRN01000569.1	981	1162	+	UREA	5' UTR	Type I
Emergomyces pasteurianus	LGRN01000020.1	51752	51580	-	THI4	5' UTR	Type I
Emergomyces pasteurianus	LGRN01000162.1	27480	27683	+	NMT1	5' UTR	Type I
Exophiala dermatitidis	NW_008751649.1	600314	600166	-	UREA TRANSPORTER	INTRON	Type II
Exophiala dermatitidis	NW_008751647.1	3214229	3214379	+	THI4	5' UTR	Type I
Exophiala spinifera	NW_015622514.1	2694173	2694373	+	UREA TRANSPORTER	INTRON	Type II
Exophiala spinifera	NW_015622517.1	2841579	2841403	-	NMT1	5' UTR	Type I
Exophiala spinifera	NW_015622513.1	4628311	4628526	+	THI4	5' UTR	Type I
Fonsecaea monophora	NW_019173992.1	142186	142344	+	UREA TRANSPORTER	INTRON	Type II
Fonsecaea monophora	NW_019173933.1	835029	835203	+	THI4	5' UTR	Type I
Fonsecaea nubica	NW_019173742.1	106420	106579	+	UREA TRANSPORTER	INTRON	Type II
Fonsecaea nubica	NW_019173814.1	36964	36791	-	THI4	5' UTR	Type I
Fonsecaea pedrosoi	NW_013550615.1	428002	427844	-	UREA TRANSPORTER	INTRON	Type II
Fonsecaea pedrosoi	NW_013550612.1	1012313	1012487	+	THI4	5' UTR	Type I
Fusarium chlamydosporum	JAAVUD010000147.1	29741	29880	+	THI4	5' UTR	Type I

Fusarium chlamydosporum	JAAVUD010000117.1	63487	63306	-	NMT1	5' UTR	Type I
Fusarium chlamydosporum	JAAVUD010000029.1	26078	26240	+	UREA TRANSPORTER	5' UTR	Type I
Fusarium dimerum	JABGLY010000760.1	19541	19377	-	THI4	5' UTR	Type I
Fusarium dimerum	JABGLY010000935.1	9091	9235	+	NMT1	5' UTR	Type I
Fusarium dimerum	JABGLY010000760.1	19040	18890	-	urea	5' UTR	Type I
Fusarium equiseti	QOHM01000002	4378538	4378673	+	THI4	5' UTR	Type I
Fusarium equiseti	QOHM01000001	6120106	6120266	+	NMT1	5' UTR	Type I
Fusarium equiseti	QOHM01000005	2803961	2804122	+	UREA TRANSPORTER	INTRON	Type II
Fusarium fujikuroi	NC_036626.1	3315677	3315549	-	THI4	5' UTR	Type I
Fusarium fujikuroi	NC_036622.1	545253	545428	+	NMT1	5' UTR	Type I
Fusarium fujikuroi	NC_036627.1	1131376	1131217	-	UREA TRANSPORTER	INTRON	Type II
Fusarium incarnatum	QGDZ01000093.1	348911	349046	+	THI4	5' UTR	Type I
Fusarium incarnatum	QGDZ01000133.1	69936	69776	-	NMT1	5'UTR	Type I
Fusarium incarnatum	QGDZ01000017.1	155212	155052	-	UREA TRANSPORTER	INTRON	Type II
Fusarium oxysporum	NW_022158523.1	3285638	3285510	-	THI4	5'UTR	Type I
Fusarium oxysporum	NW_022158524.1	2967814	2967970	+	UREA TRANSPORTER	5'UTR	Type I
Fusarium oxysporum	NW_022158530.1	1026603	1026778	+	NMT1	5'UTR	Type I
Fusarium proliferatum	NW_022194800.1	543199	543071	-	THI4	5'UTR	Type I
Fusarium proliferatum	NW_022194790.1	5751871	5751696	-	NMT1	5'UTR	Type I
Fusarium proliferatum	NW_022194795.1	298649	298490	-	UREA TRANSPORTER	INTRON	Type II
Fusarium solani	NGZQ01000006.1	1838704	1838563	-	THI4	5'UTR	Type I
Fusarium solani	NGZQ01000001.1	5952487	5952671	+	NMT1	5'UTR	Type I
Fusarium solani	NGZQ01000005.1	1288552	1288395	-	UREA TRANSPORTER	INTRON	Type II
Fusarium sporotrichioides	JAHKNS010000001.1	5985832	5985975	+	THI4	5'UTR	Type I
Fusarium sporotrichioides	JAHKNS010000001.1	8271723	8271564	-	NMT1	5'UTR	Type I
Fusarium sporotrichioides	JAHKNS010000002.1	5970103	5969951	-	UREA TRANSPORTER	INTRON	Type II
Histoplasma capsulatum	NW_001813980.1	420379	420583	+	NMT1	5'UTR	Type I
Histoplasma capsulatum	NW_001813976.1	934335	934509	+	THI4	5'UTR	Type I
Histoplasma capsulatum	NW_001813982.1	4158337	4158149	-	UREA TRANSPORTER	INTRON	Type II
Hortaea werneckii	JACSRB010000282.1	1652	1501	-	NMT1	5' UTR	Type I
Hortaea werneckii	JACSRB010000014.1	139531	139638	+	NUCLEOSIDE TRANSPORTER	5' UTR	Type I
Hortaea werneckii	JACSRB010000055.1	10572	10679	+	NUCLEOSIDE TRANSPORTER	5' UTR	Type I
Hortaea werneckii	JACSRB010000111.1	40426	40571	+	UREA TRANSPORTER	INTRON	Type II
Hortaea werneckii	JACSRB010000207.1	9886	10032	+	UREA TRANSPORTER	INTRON	Type II
Hortaea werneckii	JACSRB010000070.1	108080	107922	-	NMT1	5'UTR	Type I
Hortaea werneckii	JACSRB010000002.1	199778	199617	-	THI4	INTRON	Type III
Hortaea werneckii	JACSRB010000015.1	53002	53163	+	THI4	INTRON	Type III

Hortaea werneckii	JACSRB010000067.1	79673	79531	-	AMINO ACID TRANSPORTER	INTRON	Type III
Lasiodiplodia theobromae	NW_023336486.1	469436	469600	+	UREA TRANSPORTER	INTRON	Type II
Lasiodiplodia theobromae	NW_023336449.1	158725	158524	-	NUCLEOSIDE TRANSPORTER	5' UTR	Type I
Lasiodiplodia theobromae	NW_023336544.1	118789	118987	+	NMT1	5' UTR	Type I
Lasiodiplodia theobromae	NW_023336530.1	1146636	1146755	+	THI4	5' UTR	Type I
Lomentospora prolificans	NLAX01001623.1	1113962	1114113	+	NMT1	5' UTR	Type I
Lomentospora prolificans	NLAX01000095.1	266710	266874	+	THI4	5' UTR	Type I
Lomentospora prolificans	NLAX01000010.1	2485831	2485607	-	UREA TRANSPORTER	INTRON	Type II
Madurella mycetomatis	LCTW02000335.1	15281	15149	-	THI4	INTRON	Type II
Madurella mycetomatis	LCTW02000011.1	111680	111528	-	UREA TRANSPORTER	INTRON	Type II
Madurella mycetomatis	LCTW02000250.1	43939	44100	+	NMT1	5' UTR	Type I
Magnusiomyces capitatus	UIDF01000002.1	1947157	1947257	+	UREA TRANSPORTER	5' UTR	Type I
Magnusiomyces clavatus	CBXB010000036.1	232735	232618	-	UREA TRANSPORTER	5' UTR	Type I
Meyerozyma guilliermondii	NW_001809798.1	919875	919762	-	UREA TRANSPORTER	5' UTR	Type I
Microsporum canis	NW_003299163.1	999342	999485	+	NMT1	5' UTR	Type I
Microsporum canis	NW_003299161.1	336661	336472	-	THI4	5' UTR	Type I
Microsporum canis	NW_003299162.1	438744	438596	-	UREA TRANSPORTER	INTRON	Type II
Nannizzia gypsea	NW_003345198.1	2247454	2247294	-	UREA TRANSPORTER	INTRON	Type II
Nannizzia gypsea	NW_003345198.1	1389615	1389470	-	NMT1	5' UTR	Type I
Nannizzia gypsea	NW_003345192.1	370560	370396	-	THI4	5' UTR	Type I
Neoscytalidium dimidiatum	LT599057.1	680841	681013	+	UREA TRANSPORTER	INTRON	Type II
Neoscytalidium dimidiatum	LT599068.1	334599	334803	+	NMT1	5' UTR	Type I
Neoscytalidium dimidiatum	LT599060.1	926622	926839	+	NUCLEOSIDE TRANSPORTER	5' UTR	Type I
Neoscytalidium dimidiatum	LT599055.1	1084858	1085018	+	THI4	5' UTR	Type I
Neurospora crassa	NC_026502.1	4177854	4178032	+	NMT1	5' UTR	Type I
Neurospora crassa	NC_026507.1	1766295	1766167	-	THI4	5' UTR	Type I
Neurospora crassa	NC_026501.1	1801551	1801344	-	UREA TRANSPORTER	5' UTR	Type II
Ochroconis constricta	AZYM01000159.1	97891	98024	+	AMINO ACID TRANSPORTER	5' UTR	Type I
Paecilomyces variotii	NW_021167140.1	872142	872233	+	PUTATIVE CHOLINESTERASE	5' UTR	Type I
Paecilomyces variotii	NW_021167141.1	3703159	3702943	-	NUCLEOSIDE TRANSPORTER	5' UTR	Type I
Paecilomyces variotii	NW_021167154.1	375836	375928	+	NMT1	5' UTR	Type I
Paecilomyces variotii	NW_021167158.1	64276	64084	-	THI4	INTRON	Type III
Paracoccidioides brasiliensis	NW_011371370.1	609619	609824	+	NMT1	5' UTR	Type I
Paracoccidioides brasiliensis	NW_011371361.1	649146	648967	-	THI4	5' UTR	Type I

Paracoccidioides brasiliensis	NW_011371364.1	1295568	1295747	+	UREA TRANSPORTER	INTRON	Type II
Paracoccidioides lutzii	NW_015440963.1	158155	157956	-	NMT1	5' UTR	Type I
Paracoccidioides lutzii	NW_015440948.1	1488959	1489132	+	THI4	INTRON	Type III
Paracoccidioides lutzii	NW_015440962.1	228156	227977	-	UREA TRANSPORTER	INTRON	Type II
Penicillium marneffei	NW_002196661.1	5219734	5219616	-	NMT1	INTRON	Type III
Penicillium marneffei	NW_002196662.1	5219733	5219617	+	THI4	INTRON	Type III
Penicillium marneffei	NW_002196665.1	1494098	1494276	+	NUCLEOSIDE TRANSPORTER	INTRON	Type III
Phialophora verrucosa	MSED01000004.1	1539867	1539702	-	THI4	5'UTR	Type I
Phialophora verrucosa	MSED01000013.1	470653	470505	-	UREA TRANSPORTER	INTRON	Type II
Pichia kudriavzevii	NC_042508.1	338398	338497	+	UREA TRANSPORTER	INTRON	Type II
Pichia norvegensis	PPIO01000029.1	102676	102764	+	UREA TRANSPORTER	INTRON	Type II
Purpureocillium lilacinum	NW_017264038.1	454726	454531	-	THI4	5'UTR	Type I
Purpureocillium lilacinum	NW_017264031.1	2255613	2255456	-	NMT1	5'UTR	Type I
Purpureocillium lilacinum	NW_017264030.1	1926272	1926418	+	UREA TRANSPORTER	INTRON	Type II
Rhinocladiella mackenziei	NW_013550603.1	2475792	2475940	+	UREA TRANSPORTER	INTRON	Type II
Rhinocladiella mackenziei	NW_013550604.1	127980	127816	-	THI4	INTRON	Type III
Scedosporium apiospermum	NW_015971795.1	519238	519394	+	NMT1	5' UTR	Type I
Scedosporium apiospermum	NW_015971787.1	1138671	1138480	-	THI4	5' UTR	Type I
Scedosporium apiospermum	NW_015971855.1	216666	216432	-	UREA TRANSPORTER	INTRON	Type II
Scedosporium aurantiacum	JUDQ01000181.1	11439	11285	-	NMT1	5' UTR	Type I
Scedosporium aurantiacum	JUDQ01000055.1	18236	18423	+	THI4	5' UTR	Type I
Scedosporium aurantiacum	JUDQ01000109.1	57518	57287	-	UREA TRANSPORTER	INTRON	Type II
Scedosporium boydii	NJFT01000193.1	19736	19892	+	NMT1	5' UTR	Type I
Scedosporium boydii	NJFT01000161.1	4029	3842	-	THI4	5' UTR	Type I
Scedosporium boydii	NJFT01000433.1	5854	5620	-	UREA TRANSPORTER	INTRON	Type II
Sporothrix schenckii	NW_015971142.1	295146	295305	+	THI4	5' UTR	Type I
Sporothrix schenckii	NW_015971139.1	1383227	1383435	+	UREA TRANSPORTER	INTRON	Type II
Sporothrix brasiliensis	NW_024467136.1	320685	320844	+	THI4	5' UTR	Type I
Sporothrix brasiliensis	NW_024467140.1	1905956	1905748	-	UREA TRANSPORTER	INTRON	Type II
Sporothrix globosa	LVYW01000004.1	4249204	4249053	-	THI4	5' UTR	Type IV
Sporothrix globosa	LVYW01000006.1	1880916	1880706	-	UREA TRANSPORTER	INTRON	Type II
Stachybotrys chartarum	LDEE01000005.1	2064734	2064591	-	NUCLEOSIDE TRANSPORTER	5' UTR	Type II
Stachybotrys chartarum	LDEE01000018.1	320656	320817	+	THI4	5' UTR	Type I
Stachybotrys chartarum	LDEE01000006.1	1321088	1321190	+	UREA TRANSPORTER	INTRON	Type II

Stachybotrys chartarum	LDEE01000003.1	1639905	1639760	-	NMT1	5' UTR	Type I
Talaromyces marneffei	NW_002196661.1	5219734	5219616	-	NMT1	INTRON	Type III
Talaromyces marneffei	NW_002196662.1	3088045	3088171	+	THI4	INTRON	Type III
Talaromyces marneffei	NW_002196665.1	1494098	1494276	+	NUCLEOSIDE TRANSPORTER	INTRON	Type II
Thermothielavioides terrestris	NC_016459.1	3078693	3078865	+	NMT1	5' UTR	Type I
Thermothielavioides terrestris	NC_016459.1	2884190	2884034	-	THI4	5' UTR	Type I
Thermothielavioides terrestris	NC_016458.1	855263	855426	+	UREA TRANSPORTER	INTRON	Type II
Trichoderma harzianum	NW_020209253.1	831953	831770	-	NMT1	5' UTR	Type I
Trichoderma harzianum	NW_020209261.1	186981	186771	-	THI4	5' UTR	Type I
Trichoderma harzianum	NW_020209249.1	1933278	1933121	-	UREA TRANSPORTER	INTRON	Type II
Trichoderma longibrachiatum	KB290584.1	1035067	1034873	-	THI4	5' UTR	Type I
Trichoderma longibrachiatum	KB290592.1	84749	84569	-	NMT1	5' UTR	Type I
Trichoderma longibrachiatum	ANBJ01000133.1	22475	22302	-	UREA TRANSPORTER	INTRON	Type II
Trichoderma citrinoviride	NW_020194716.1	584818	584632	-	THI4	5' UTR	Type I
Trichoderma citrinoviride	NW_020194725.1	102324	102499	+	NMT1	5' UTR	Type I
Trichoderma citrinoviride	NW_020194709.1	1351958	1351771	-	UREA TRANSPORTER	5' UTR	Type II
Trichoderma viride	VCEC01000017.1	339124	338924	-	THI4	5' UTR	Type I
Trichoderma viride	VCEC01000005.1	565002	564820	-	NMT1	5' UTR	Type I
Trichoderma viride	VCEC01000003.1	2261621	2261761	+	UREA TRANSPORTER	INTRON	Type II
Trichophyton equinum	DS995740.1	95127	94973	-	THI4	5' UTR	Type I
Trichophyton equinum	DS995718.1	149827	149994	+	NMT1	5' UTR	Type I
Trichophyton equinum	DS995742.1	356180	356026	-	UREA TRANSPORTER	INTRON	Type II
Trichophyton interdigitale	JAADCK010000011.1	151908	152067	+	THI4	5' UTR	Type I
Trichophyton interdigitale	JAADCK010000001.1	85401	85568	+	NMT1	5' UTR	Type I
Trichophyton interdigitale	JAADCK010000079.1	33468	33622	+	UREA TRANSPORTER	INTRON	Type II
Trichophyton mentagrophytes	QQSR01000003.1	59242	59401	+	THI4	5' UTR	Type I
Trichophyton mentagrophytes	QQSR01000008.1	50421	50588	+	NMT1	5' UTR	Type I
Trichophyton mentagrophytes	QQSR01000036.1	115072	114918	-	UREA TRANSPORTER	INTRON	Type II
Trichophyton rubrum	NW_003456426.1	220484	220644	+	THI4	5' UTR	Type I
Trichophyton rubrum	NW_003456427.1	1374470	1374298	-	NMT1	5' UTR	Type I
Trichophyton rubrum	NW_003456427.1	2210428	2210274	-	UREA TRANSPORTER	INTRON	Type II
Trichophyton soudanense	CAJUYN010000016.1	218350	218510	+	THI4	5' UTR	Type I
Trichophyton soudanense	CAJUYN010000017.1	1386913	1386741	-	NMT1	5' UTR	Type I

Trichophyton soudanense	CAJUYN010000017.1	2216278	2216124	-	UREA TRANSPORTER	INTRON	Type II
Trichophyton tonsurans	GG698511.1	27270	27111	-	THI4	5' UTR	Type I
Trichophyton tonsurans	GG698492.1	266059	265892	-	NMT1	5' UTR	Type I
Trichophyton tonsurans	GG698488.1	451541	451387	-	UREA TRANSPORTER	INTRON	Type II
Trichophyton verrucosum	NW_003315429.1	40157	39997	-	THI4	5' UTR	Type I
Trichophyton verrucosum	NW_003315393.1	31237	31408	+	NMT1	5' UTR	Type I
Trichophyton verrucosum	NW_003315532.1	96763	96921	+	UREA TRANSPORTER	INTRON	Type II
Trichophyton violaceum	CAJUYU010000016.1	222626	222786	+	THI4	5' UTR	Type I
Trichophyton violaceum	CAJUYU010000017.1	1357116	1356944	-	NMT1	5' UTR	Type I
Trichophyton violaceum	CAJUYU010000017.1	2233247	2233093	-	UREA TRANSPORTER	INTRON	Type II
Trichothecium roseum	PXNY01000089.1	68108	68249	+	UREA TRANSPORTER	INTRON	Type II
Trichothecium roseum	PXNY01000056.1	107909	108074	+	THI4	5' UTR	Type I
Trichothecium roseum	PXNY01000406.1	17282	17088	-	NMT1	5' UTR	Type I
Verruconis gallopava	NW_015622137.1	145789	145917	+	NUCLEOSIDE TRANSPORTER	INTRON	Type III
Verruconis gallopava	NW_015622144.1	715293	715154	-	AMINO ACID TRANSPORTER	INTRON	Type III
Verticillium longisporum	JAETXT010000015.1	1308972	1309118	+	NMT1	INTRON	Type III
Verticillium longisporum	JAETXT010000007.1	270650	270796	+	NMT1	INTRON	Type III
Verticillium longisporum	JAETXT010000002.1	2726107	2726289	+	THI4	5' UTR	Type I
Verticillium longisporum	JAETXT010000001.1	1019596	1019774	+	THI4	5' UTR	Type I
Verticillium longisporum	JAETXT010000001.1	6822014	6822156	+	UREA TRANSPORTER	INTRON	Type II
Verticillium longisporum	JAETXT010000003.1	4186207	4186349	+	UREA TRANSPORTER	INTRON	Type II
Verticillium albo-atrum	NMXJ01000007.1	217159	217013	-	NMT1	INTRON	Type III
Verticillium albo-atrum	NMXJ01000003.1	1904618	1904795	+	THI4	INTRON	Type III
Verticillium albo-atrum	NMXJ01000001.1	5171244	5171402	+	UREA TRANSPORTER	INTRON	Type II
Verticillium tricorpus	JPET01000004.1	3901619	3901764	+	NMT1	INTRON	Type III
Verticillium tricorpus	JPET01000002.1	6564731	6564547	-	THI4	INTRON	Type III
Verticillium tricorpus	JPET01000002.1	1657101	1657248	+	UREA TRANSPORTER	INTRON	Type II
Wickerhamomyces anomalus	NW_017567111.1	536997	537097	+	UREA TRANSPORTER	INTRON	Type II
Verticillium dahliae	NW_009276935.1	87576	87430	-	NMT1	INTRON	Type III
Verticillium dahliae	NW_009276916.1	1009464	1009642	+	THI4	INTRON	Type III
Verticillium dahliae	NW_009276925.1	382365	382223	-	UREA TRANSPORTER	INTRON	Type II
Yarrowia lipolytica	NC_006070.1	2512532	2512607	+	UREA TRANSPORTER	5' UTR	Type IV
Yarrowia lipolytica	NC_006071.1	3315134	3315257	+	SODIUM-DEPENDENT TRANSPORTER	5' UTR	Type I

Coprinopsis cinerea	NW_003307540.1	820070	819963	-	AMINO ACID TRANSPORTER	INTRON	Type III
Cryptococcus albidus	BCHV01000035.1	29462	29321	-	AMINO ACID TRANSPORTER	INTRON	Type I
Cryptococcus albidus	BCHV01000020.1	134723	134548	-	GABA transporter	5' UTR	Type I
Cryptococcus gattii VGI	NC_014944.1	145186	145073	-	AMINO ACID TRANSPORTER	5' UTR	Type I
Cryptococcus gattii VGI	NC_014943.1	1242037	1241942	-	GABA transporter	5' UTR	Type I
Cryptococcus gattii VGIII	KN848891.1	1139611	1139745	+	AMINO ACID TRANSPORTER	5' UTR	Type I
Cryptococcus gattii VGIII	KN848894.1	93253	93138	-	GABA transporter	5' UTR	Type I
Cryptococcus gattii VGIV	KN848856.1	145361	145227	-	AMINO ACID TRANSPORTER	5' UTR	Type I
Cryptococcus gattii VGIV	KN848862.1	67498	67591	+	GABA transporter	5' UTR	Type I
Cryptococcus laurentii	JAAZPX010000030.1	461091	460940	-	SUGAR TRANSPORTER	INTRON	Type III
Cryptococcus neoformans	NC_006692.1	1194184	1194302	+	AMINO ACID TRANSPORTER	INTRON	Type III
Cryptococcus neoformans	NC_006691.1	114957	114863	-	GABA transporter	INTRON	Type III
Cutaneotrichosporon dermatis	JAIGNX010000014.1	123104	122979	-	SUGAR TRANSPORTER	5'UTR	Type I
Malassezia furfur	CP046234.1	66521	66338	+	GTP-BINDING PROTEIN	5'UTR	Type IV
Malassezia furfur	CP046234.1	2873185	2873315	+	UREA TRANSPORTER	5'UTR	Type IV
Phanerochaete sordida	BPQB01000001.1	2084821	2084995	+	AMINO ACID TRANSPORTER	INTRON	Type III
Phanerodontia chrysosporium	MJGA01000001.1	1017200	1017046	-	AMINO ACID TRANSPORTER	5'UTR	Type I
Rhodotorula mucilaginosa	PEFX01000025.1	388347	388461	+	THI4	INTRON	Type III
Rhodotorula mucilaginosa	PEFX01000009.1	889664	889570	-	AMINO ACID TRANSPORTER	INTRON	Type III
Schizophyllum commune	NW_003315651.1	22993	23103	+	AMINO ACID TRANSPORTER	5' UTR	Type I
Trichosporon asahii	NW_014040899.1	185577	185696	+	SUGAR TRANSPORTER	5' UTR	Type I
Trichosporon cutaneum	BCKU01000028.1	165904	165797	-	SUGAR TRANSPORTER	INTRON	Type III
Trichosporon coremiiforme	JXYL01000003.1	125275	125146	-	SUGAR TRANSPORTER	INTRON	Type III
Trichosporon coremiiforme	JXYL01000007.1	1588296	1588173	-	SUGAR TRANSPORTER	INTRON	Type III
Trichosporon inkin	JXYM01000002.1	2260007	2259861	-	SUGAR TRANSPORTER	INTRON	Type III
Trichosporon mucoides	BCJT01000002.1	2902011	2902133	+	SUGAR TRANSPORTER	5' UTR	Type I
Trichosporon mucoides	BCJT01000006.1	111094	110972	-	SUGAR TRANSPORTER	INTRON	Type III
Trichosporon ovoides	JXYN01000016.1	168325	168191	-	SUGAR TRANSPORTER	INTRON	Type III
Trichosporon ovoides	JXYN01000002.1	2933491	2933640	+	SUGAR TRANSPORTER	INTRON	Type III
Volvariella volvacea	KB722768.1	303067	302908	-	AMINO ACID TRANSPORTER	INTRON	Type III
Actinomucor elegans	BCHK01000001.1	1126136	1126253	+	NMT1	5' UTR	Type I
Actinomucor elegans	BCHK01000003.1	1225569	1225665	+	SUGAR TRANSPORTER	5' UTR	Type I
Actinomucor elegans	BCHK01000004.1	904576	904680	+	THI4	5' UTR	Type I

Actinomucor elegans	BCHK01000064.1	36792	36700	-	SUGAR TRANSPORTER	INTRON	Type III
Apophysomyces elegans	JNDQ01001525.1	44867	44985	+	NMT1	5' UTR	Type I
Apophysomyces elegans	JNDQ01001490.1	267926	268022	+	THI4	5' UTR	Type I
Apophysomyces ossiformis	JABAYA010000015.1	213218	213104	-	NMT1	5' UTR	Type I
Apophysomyces ossiformis	JABAYA010000198.1	14150	14045	-	THI4	5' UTR	Type I
Apophysomyces trapeziformis	JNDP01001000.1	154523	154410	-	NMT1	5' UTR	Type I
Apophysomyces trapeziformis	JNDP01001052.1	197756	197860	+	THI4	5' UTR	Type I
Apophysomyces variabilis	MZZL01000409.1	2207679	2207566	-	NMT1	INTRON	Type III
Apophysomyces variabilis	MZZL01000398.1	541230	541126	-	THI4	INTRON	Type III
Cokeromyces recurvatus	JNEH01001536.1	18826	18708	-	SUGAR TRANSPORTER	5' UTR	Type I
Cokeromyces recurvatus	JNEH01002304.1	12012	12107	+	SUGAR TRANSPORTER	5' UTR	Type I
Cunninghamella bertholletiae	JNEG01000657.1	15230	15127	-	THI4	5' UTR	Type I
Cunninghamella bertholletiae	JNEG01000810.1	306439	306546	+	SUGAR TRANSPORTER	5' UTR	Type I
Cunninghamella blakesleeana	JAFCIZ010000011.1	545753	545612	-	NMT1	5' UTR	Type I
Cunninghamella blakesleeana	JAFCIZ010000005.1	5514874	5514989	+	THI4	5' UTR	Type I
Cunninghamella blakesleeana	JAFCIZ010000004.1	684542	684444	-	SUGAR TRANSPORTER	INTRON	Type III
Cunninghamella blakesleeana	JAFCIZ010000005.1	5037398	5037297	-	SUGAR TRANSPORTER	INTRON	Type III
Cunninghamella elegans	JNDR01001308.1	389649	389561	-	THI4	5' UTR	Type I
Cunninghamella elegans	JNDR01001328.1	69611	69525	-	SUGAR TRANSPORTER	INTRON	Type III
Cunninghamella elegans	JNDR01001273.1	304372	304235	-	NMT1	5' UTR	Type I
Cunninghamella elegans	JNDR01001226.1	151213	151298	+	SUGAR TRANSPORTER	5' UTR	Type I
Lichtheimia corymbifera	JNEU01000982.1	76851	76967	+	AMINO ACID TRANSPORTER	5' UTR	Type I
Mucor circinelloides	BCHG01000036.1	211291	211173	-	NMT1	5' UTR	Type I
Mucor circinelloides	BCHG01000176.1	42032	41925	-	THI4	5' UTR	Type I
Mucor circinelloides	BCHG01000121.1	71882	71773	-	THI4	5' UTR	Type I
Mucor circinelloides	BCHG01000097.1	112005	112123	+	SUGAR TRANSPORTER	5' UTR	Type I
Mucor circinelloides	BCHG01000203.1	24230	24151	-	SUGAR TRANSPORTER	5' UTR	Type I
Mucor indicus	JNEK01002716.1	113526	113398	-	SUGAR TRANSPORTER	5' UTR	Type I
Mucor indicus	JNEK01002813.1	1740	1633	-	THI4	5' UTR	Type I
Mucor indicus	JNEK01001413.1	44867	44947	+	SUGAR TRANSPORTER	5' UTR	Type I
Mucor irregularis	AZYI01000160.1	82877	82745	-	NMT1	5' UTR	Type I
Mucor irregularis	KK076499.1	93518	93399	-	SUGAR TRANSPORTER	INTRON	Type III
Mucor irregularis	KK076482.1	322425	322323	-	SUGAR TRANSPORTER	INTRON	Type III
Mucor irregularis	AZYI01000166.1	111056	110950	-	THI4	5' UTR	Type I

Mucor irregularis	KK076457.1	13469	13380	-	SUGAR TRANSPORTER	5' UTR	Type I
Mucor lusitanicus	JAAECE010000005.1	1696354	1696237	-	ATP binding protein	5' UTR	Type I
Mucor lusitanicus	JAAECE010000006.1	2508737	2508838	+	ATP binding protein	5' UTR	Type I
Mucor lusitanicus	JAAECE010000003.1	3203555	3203449	-	SUGAR TRANSPORTER	5' UTR	Type I
Mucor lusitanicus	JAAECE010000004.1	23025	22946	-	SUGAR TRANSPORTER	5' UTR	Type I
Mucor racemosus	JNEI01000064.1	59	169	+	ATP binding protein	INTRON	Type III
Mucor racemosus	JNEI01001054.1	13531	13411	-	ATP binding protein	5' UTR	Type I
Mucor racemosus	JNEI01002594.1	15926	16034	+	THI4	5' UTR	Type I
Mucor racemosus	JNEI01005443.1	9539	9433	-	THI4	INTRON	Type III
Mucor racemosus	JNEI01004296.1	16569	16697	+	SUGAR TRANSPORTER	5' UTR	Type I
Mucor racemosus	JNEI01004774.1	10300	10384	+	SUGAR TRANSPORTER	5' UTR	Type I
Mucor velutinosus	JNDK01001171.1	6724	6624	-	NMT1	INTRON	Type III
Mucor velutinosus	JNDK01001242.1	46079	46184	+	THI4	INTRON	Type III
Mucor velutinosus	JNDK01002029.1	11162	11251	+	THI4	5' UTR	Type I
Mucor velutinosus	JNDK01002078.1	79599	79708	+	SUGAR TRANSPORTER	5' UTR	Type I
Mucor velutinosus	JNDK01002401.1	32755	32834	+	SUGAR TRANSPORTER	5' UTR	Type I
Rhizomucor miehei	KK100182.1	208164	208211	+	SUGAR TRANSPORTER	5' UTR	Type I
Rhizomucor miehei	KK100222.1	12359	12494	+	SUGAR TRANSPORTER	5' UTR	Type I
Rhizomucor pusillus	FWWN02000668.1	172873	172772	-	NMT1	5' UTR	Type I
Rhizomucor pusillus	FWWN02000389.1	50823	50747	-	SUGAR TRANSPORTER	5' UTR	Type I
Rhizomucor pusillus	FWWN02000573.1	29772	29942	+	SUGAR TRANSPORTER	5' UTR	Type I
Rhizopus oryzae	JNEF01002634.1	11464	11384	-	SUGAR TRANSPORTER	INTRON	Type III
Rhizopus oryzae	JNEF01003205.1	25173	25260	+	SUGAR TRANSPORTER	5' UTR	Type I
Rhizopus oryzae	JNEF01002758.1	63191	63290	+	THI4	5' UTR	Type I
Rhizopus oryzae	JNEF01002627.1	6953	6821	-	NMT1	INTRON	Type III
Rhizopus microsporus	NW_019671916.1	2772802	2772697	-	THI4	INTRON	Type III
Rhizopus microsporus	NW_019671946.1	97484	97400	-	SUGAR TRANSPORTER	5' UTR	Type I
Rhizopus microsporus	NW_019671927.1	257522	257389	-	NMT1	5' UTR	Type I
Rhizopus stolonifer	JNDS01005489.1	26416	26336	-	SUGAR TRANSPORTER	INTRON	Type III
Rhizopus stolonifer	JNDS01005275.1	23362	23256	-	NMT1	5' UTR	Type I
Rhizopus stolonifer	JNDS01005479.1	24151	24259	+	THI4	5' UTR	Type I
Saksenaea vasiformis	JNDT01001565.1	42397	42284	-	NMT1	INTRON	Type III
Saksenaea vasiformis	JNDT01001847.1	37265	37369	+	THI4	5' UTR	Type I
Saksenaea oblongispora	JNEV01001177.1	64810	64697	-	NMT1	5' UTR	Type I
Saksenaea oblongispora	JNEV01001638.1	11298	11402	+	THI4	INTRON	Type III
Syncephalastrum racemosum	MCGN01000013.1	589130	589225	+	NMT1	5' UTR	Type I
Syncephalastrum racemosum	MCGN01000008.1	1476204	1476310	+	THI4	5' UTR	Type I

Syncephalastrum racemosum	MCGN01000011.1	784421	784306	-	SUGAR TRANSPORTER	INTRON	Type III

*A complete version of this table, including the taxonomic classification, GC content, and size of each sequence, is provided as a separate XLSX file.
